# Earthworm‐Inspired Ultra‐Durable Sliding Triboelectric Nanogenerator with Bionic Self‐Replenishing Lubricating Property for Wind Energy Harvesting and Self‐Powered Intelligent Sports Monitoring

**DOI:** 10.1002/advs.202401636

**Published:** 2024-05-13

**Authors:** Mengjiao Liu, Xin Zhang, Yue Xin, Dongxu Guo, Guangkai Hu, Yifei Ma, Bin Yu, Tao Huang, Chengchang Ji, Meifang Zhu, Hao Yu

**Affiliations:** ^1^ State Key Laboratory for Modification of Chemical Fibers and Polymer Materials, College of Materials Science and Engineering Donghua University Shanghai 201620 China; ^2^ College of Information Science and Technology Donghua University Shanghai 201620 China; ^3^ College of Computer Science and Technology Donghua University Shanghai 201620 China; ^4^ Lehrstuhl für Chemische Reaktionstechnik Friedrich‐Alexander‐Universität Erlangen‐Nürnberg 91058 Erlangen Germany

**Keywords:** bionic self‐replenishing lubricating, intelligent sports monitoring, sliding triboelectric nanogenerator, ultra‐durable, wind energy harvesting

## Abstract

Triboelectric nanogenerators (TENGs), a promising strategy for harvesting distributed low‐quality power sources, face inevitable bottlenecks regarding long‐term abrasion and poor durability. Herein, both issues are addressed by selecting an earthworm‐inspired self‐replenishing bionic film (ERB) as the tribo‐material of sliding‐freestanding TENGs (SF‐TENGs), it consists of an interconnected 3D porous network structure capable of storing and releasing lubricant under cyclic mechanical stimuli. Thanks to the superiority of self‐replenishing property, there is no need for periodic replenishment and accurate content control of lubricant over the interfacial‐lubricating SF‐TENGs based on dense tribo‐layers. Additionally, an SF‐TENG based on ERB film (ERB‐TENG) demonstrates remarkable output stability with only a slight attenuation of 1% after continuous operation for 100 000 cycles. Moreover, the ERB‐TENG displays a distinguished anti‐wear property, exhibiting no distinct abrasion with an ultra‐low coefficient of friction (0.077) and maintaining output stability over a prolonged period of 35 days. Furthermore, integration with an energy management circuit enables the ERB‐TENG to achieve a 39‐fold boost in charging speed. This work proposes a creative approach to enhance the durability and extend the lifespan of TENG devices, which is also successfully applied to wind energy harvesting and intelligent sports monitoring.

## Graphical abstract

Inspired by the physiological behavior of earthworms, an innovative ultra‐durable sliding‐freestanding triboelectric nanogenerator (SF‐TENG) is designed, featuring a self‐replenishing lubricating property. It is endowed with remarkable wear resistance and outstanding output stability, with only a 1% attenuation after consecutive 100 000 cycles. This work proposes an intriguing and pioneering strategy for extending the lifespan and application possibilities of SF‐TENGs.

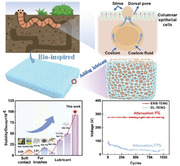

## 1 Introduction

With the escalating issues of global climate change and the unsustainability of fossil energy, achieving carbon neutrality has become a consensus among the international community.^[^ ^1^, ^2^ ^]^ Conventional power supply approaches face significant challenges in meeting the increasing demand for distributed energy supply, particularly for ubiquitous electronic devices. Consequently, there is an urgent need to develop innovative energy supply systems to facilitate the development of smart, pervasive, and energy‐efficient electronics. As a novel type of energy harvesting and conversion system, triboelectric nanogenerators (TENGs) based on Maxwell displacement current have shown great promise in converting a variety of low‐quality mechanical energy into electricity.^[^ ^3^, ^4^, ^5^ ^]^ Among them, sliding‐freestanding TENGs (SF‐TENGs) have gained considerable attention from researchers, as they provide high energy conversion efficiency.^[^ ^6^ ^]^ Nevertheless, the matter of material abrasion caused by in‐plane intense friction severely hinders the further development of SF‐TENGs.^[^ ^7^, ^8^, ^9^ ^]^

Hence, large efforts have been dedicated to prolonging the service life and heightening the robustness of SF‐TENGs utilizing structural optimization,^[^ ^10^, ^11^, ^12^, ^13^ ^]^ introducing fur brushes,^[^ ^14^, ^15^, ^16^ ^]^ interfacial lubrication,^[^ ^17^, ^18^, ^19^, ^20^, ^21^, ^22^ ^]^ and so on. From the perspective of structural optimization, this strategy is not well suitable for further enhancing the output performance of SF‐TENGs due to their inherent device structural design that sacrifices contact area. To improve the durability and long‐term stability of SF‐TENGs on the premise of maintaining high output performance, several studies have focused on material optimization by introducing fur brushes to lessen frictional resistance without compromising the contact area.^[^ ^15^ ^]^ Nonetheless, fur brushes encounter obstacles such as limited availability of fur sources as well as uncontrollable fur thickness and density. Currently, interfacial liquid lubrication has emerged as the mainstream approach to decrease friction and abrasion among the tribo‐pairs while ensuring sufficient contact intimacy between triboelectric layers.^[^ ^23^ ^]^ Wu et al. introduced a liquid lubricant on the surface of PI‐dense film to enhance the wear resistance of SF‐TENGs and regulated its output performance by screening the type of lubricant.^[^ ^24^ ^]^ Zhou et al. used squalane as the lubricant to obtain a high output and durable SF‐TENG by optimizing its content.^[^ ^25^ ^]^ However, a notable concern arises from the fact that the lubricant on the surface of the triboelectric dense films involved in the above reports is gradually consumed during sliding in all probability, thus necessitating periodic replenishment to ensure optimal electric performance and lubricating property. In addition, the content of lubricant is supposed to be controlled with great precision. Once the lubricant is excessive, it will significantly restrain the output performance of SF‐TENGs, thereby hindering their practical applications. Besides, the long‐term placement stability of liquid lubricant on the triboelectric film is not yet known. These matters highlight the need for further optimizing tribo‐materials of SF‐TENGs to address the aforementioned limitations for enhancing their longevity.

As is well known, earthworms possess an extraordinary ability to navigate through dry soil without causing injury. This can be put down to their sophisticated epidermal glands that can continually secrete mucus from dorsal pores and columnar epithelial cells in response to external mechanical stimuli. Moreover, their rough skin, consisting of macroscopic annuli and micro ripples, stabilizes the secreted mucus, thereby forming a thick lubricative layer for drag reduction and anti‐wear.^[^ ^26^ ^]^ Inspired by earthworms, we propose designing a hierarchical porous structure that can store lubricant using capillary effect and discharge it from holes with external mechanical stimulation, aiming to achieve an ultra‐durable triboelectric material and address the challenges of lubricant loss, poor stability, and the need for precise control of content.

Herein, we construct an earthworm‐inspired self‐replenishing bionic film (ERB) that forms an interconnected 3D porous network structure. The film possesses the characteristic of self‐replenishing lubricating when subjected to mechanical stimuli, enabling the creation of an SF‐TENG based on ERB film (ERB‐TENG) with remarkable performance, which features prominent output stability and extraordinary anti‐wear performance after continuous operation for 100 000 cycles. Importantly, precise control of the lubricant content is not required. Additionally, even if the adsorbed oil reaches the saturation point of the ERB film, it will not have a negative impact on the output performance. This is in contrast to ordinary dense films, conferring a significant enhancement in its practicality. Such outstanding merits of ERB hold considerable potential for long‐term energy harvesting and smart sports monitoring sensing domains.

## 2 Results and Discussion

### 2.1 Bionic Structure Design of ERB

**Figure 1:**
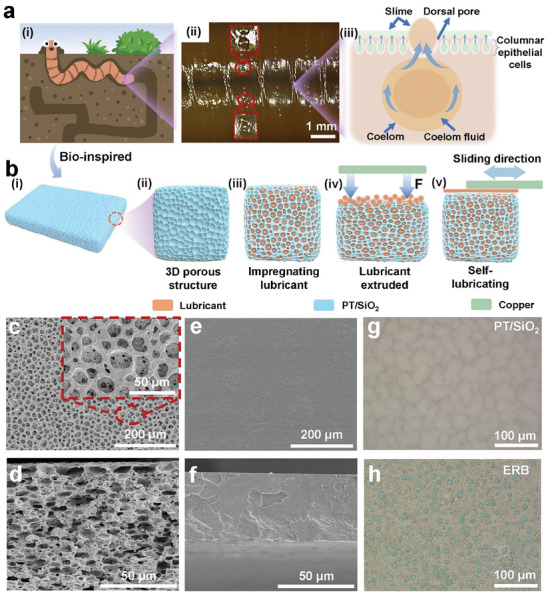
Bionic structure design and morphology characterization of ERB. a) The secretion behavior under extra stimulation: i) graphical illustration of an earthworm passing through arid soil; ii) image of earthworm's skin after mechano‐stimulus; iii) schematic for earthworm surface texture and secretion mechanism. b) Illustration of the stimuli‐responsive release of the ERB film: i) 3D structure and morphology diagram of PT/SiO_2_ film; ii) enlarged diagram of the cross‐section of PT/SiO_2_ film; iii) the PT/SiO_2_ film was immersed in a lubricant until it reached the saturation point. The lubricant was stored within the porous structure of the PT/SiO_2_ film; iv) the lubricant is extruded from the porous structure of ERB to the friction interface; v) with periodic reciprocating sliding, ERB film can achieve self‐replenishing lubrication. The scanning electron microscopy (SEM) images of c) the surface and d) cross‐section of PT/SiO_2_, respectively. SEM images show the structure of DT film from different perspectives: e) surface and f) cross‐section. Comparison of ultra depth of field images of PT/SiO_2_ g) before and h) after adding lubricant.

The lubricating mechanism of earthworms is schematically depicted in **Figure** **1**a. Earthworms possess sophisticated epidermal glands that consecutively secrete mucus in response to external mechanical stimuli. The rough skin of the earthworms enables the mucus to be stabilized, creating a slippery layer that reduces drag. This fascinating lubricating mechanism has gained significant attention for the development of low‐friction thin layers with anti‐wear and durable properties. Drawing inspiration from this lubricating mechanism, a bioinspired design is proposed to create a 3D porous structure through phase separation for self‐replenishing lubricating (Figure 1b), which is fabricated by negative tribo‐materials referred to as THV, a terpolymer comprising tetrafluoroethylene (TFE), hexafluoropropylene (HFP), and vinylidene fluoride (VDF). The resulting porous film, named PT/SiO_2_, is a porous THV (PT) film that has been modified with 0.5 wt.% oleophilic silicon dioxide nanoparticles (SiO_2_). The typical surface and cross‐section morphology of PT/SiO_2_ are shown in Figure 1c,d, respectively. The PT/SiO_2_ surface displays lunar‐like crater structures, characterized by micro‐pores in the middle. Moreover, it exhibits a 3D interconnected porous structure on the cross‐section. These features contrast sharply with the dense THV (DT) film depicted in Figure 1e,f. The ERB film with self‐replenishing lubricating is obtained by submerging the PT/SiO_2_ in squalane until it becomes saturated with oil. The ultra‐depth of field microscope images of PT/SiO_2_ (Figure 1g) and ERB (Figure 1h) film reveal a noticeable contrast. The lubricant disperses into small droplets and is uniformly distributed in the ERB film, effectively demonstrating successful lubricant storage within the film.

### 2.2 Fabrication of ERB Film and Mechanism of ERB‐TENG

**Figure 2:**
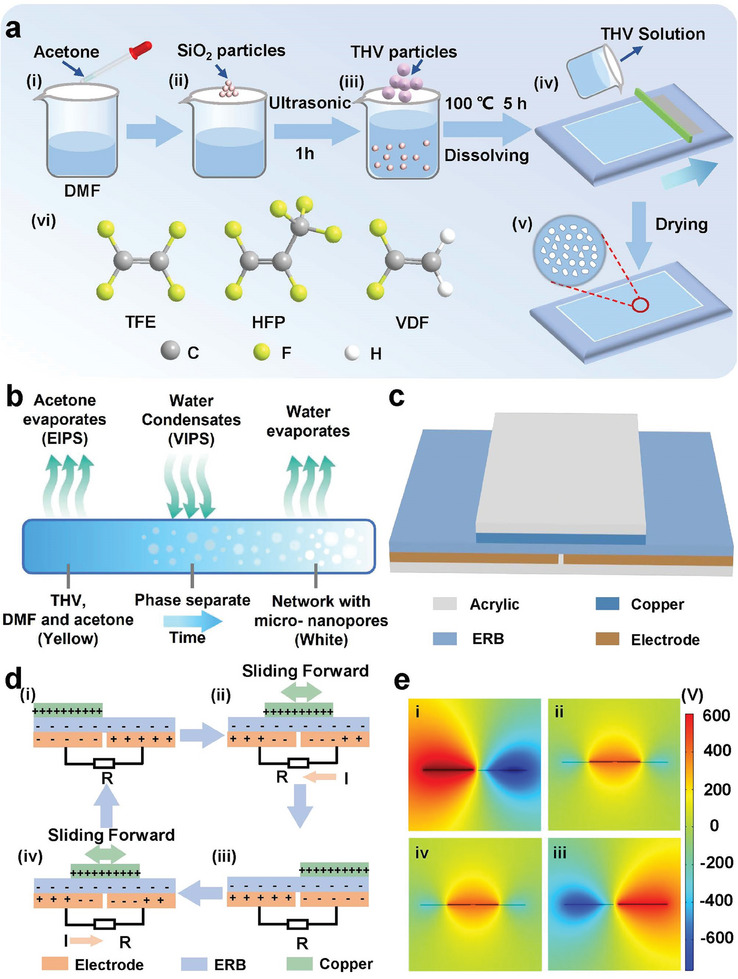
Schematic diagram of fabricating ERB films and working principle of the ERB‐TENG. a) Diagram of the synthetic strategy employed to obtain the ERB film: vi) Ball‐and‐stick models of the three monomer molecules that consist of THV material. b) Diagrammatic illustration of the methodology for investigating the dynamic formation mechanism of ERB film. c) Architecture of the ERB‐TENG. d) Charge distribution and e) simulated potential distribution of i–iv) the four states existing during a single cycle of electricity generation.

**Figure** **2**a schematically depicts the fabrication process of the ERB film. The film is typically produced using a homogeneous solution consisting of THV particles dissolved in a mixture of N,N‐Dimethylformamide (DMF) and acetone doped with SiO_2_ (Figure S1, Supporting Information). A detailed description of this process is outlined in the Experimental Section. The formation of the internal and external porous structures in the ERB film is due to the synergetic effect of vapor‐induced phase separation (VIPS) and thermally induced phase separation (EIPS),^[^ ^27^, ^28^, ^29^, ^30^, ^31^ ^]^ as illustrated in Figure 2b and Note S1 (Supporting Information). According to the formation mechanism of porous structure, two strategies are employed to achieve PT films with high porosity (Note S2, Supporting Information): altering the ratio of DMF/acetone solvent (ranging from 1:4 to 1:0.3) and adjusting the relative humidity (ranging from 33% to 88%). These strategies are advantageous for storing lubricant, as plotted in Figures S2 and S3 (Supporting Information). The experiment results revealed that a decrease in acetone content leads to the reduction of the lunar‐like crater structures and less portion of pores on the surface, but a gradual increase of pore diameter in cross‐section. A maximum porosity of 69.4% was achieved when the DMF/acetone ratio was 1:2, as provided in Figure S3 (Supporting Information). This phenomenon is ascribed to the higher acetone content which results in rapid accumulation and condensation of water vapor on the surface. This accumulation forms larger droplets that leave larger sizes of lunar‐like crater structures on the surface of films after the volatilization of water droplets. Additionally, less water vapor entered the interior of the solution, resulting in the formation of smaller‐sized internal pores. Therefore, it can be inferred that humidity will also affect its surface morphology and porosity. Figure S4 (Supporting Information) shows the surface morphology and the pore structure of the PT films fabricated in different humidity with the DMF/acetone ratio of 1:2 (w/w). It can be observed that with the increase in humidity, the size of the lunar‐like crater structures on the surface noticeably increases, while fewer pore structures are formed (Figure S4a–j, Supporting Information). However, the interior pores which showed in the cross‐section image (Figure S4k–o, Supporting Information) exhibited the opposite trend, highlighting the essential role of water vapor in pore formation. In summary, under the dual regulation of DMF/acetone (1:2) and humidity (40%), the maximum porosity obtained for the PT film is 74.5%. This provides rich pore structures for the storage of lubricants.

The device structure of the ERB‐TENG is illustrated in Figure 2c, where the ERB film and a copper foil (Cu foil, 30 × 30 mm^2^) serve as the tribo‐materials, while two conductive fabrics with the same dimensions act as symmetric electrodes. The detailed fabrication process is described in the Experimental Section. The operating principle of the ERB‐TENG is schematically presented in Figure 2d. Accompanied by the relative sliding motion between the Cu foil and the ERB layer, the electricity generation process can be universally divided into four states. The Cu foil slides between two adjacent conductive fabric electrodes: electrode A (left electrode) and electrode B (right electrode), which adhere to the ERB film. In the initial state, the Cu foil completely overlaps with electrode A (Figure 2d–i), producing positive charges on the Cu foil due to triboelectrification, equal amounts of negative charges are generated on the surface of the ERB film. As the Cu foil slides toward electrode B, positive charges flow from electrode B to A, generating a current via the external circuit to balance the change in potential difference (Figure 2d‐ii). Once the Cu foil is fully in contact with the ERB film directly above electrode B, all the positive charges are transferred to electrode A (Figure 2d‐iii). As the sliding motion continues, the positive charges flow back to electrode B (Figure 2d‐iv), inducing an inverse current. The ideal potential distributions of the two electrodes under the aforementioned four states were visualized through a finite element simulation using COMSOL software, as plotted in Figure 2e.

### 2.3 Output Performance and Durability of the ERB‐TENG

**Figure 3:**
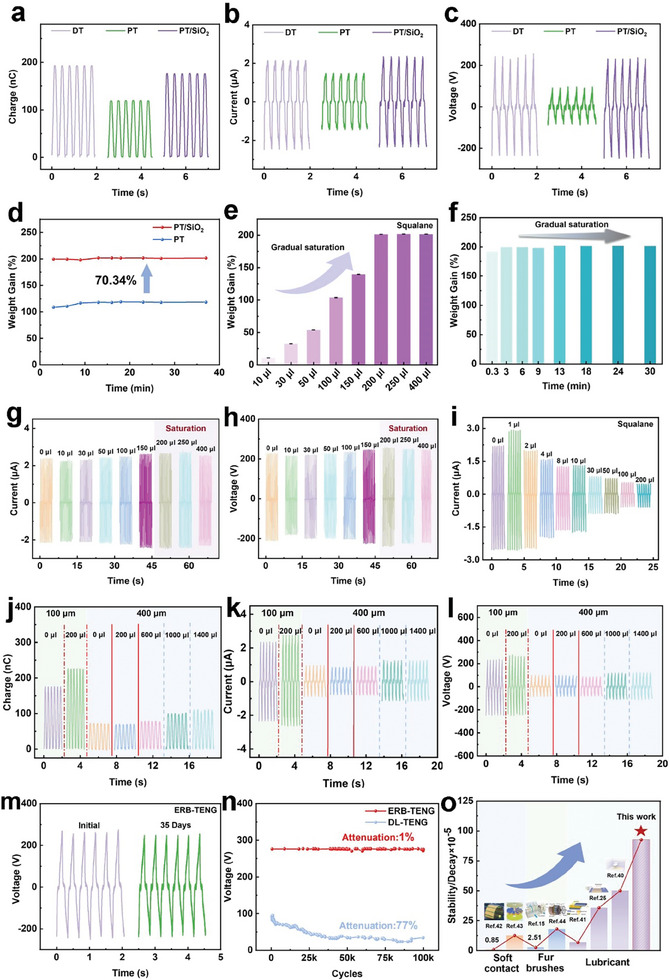
Output performance and superior durability of ERB‐TENG. a) Transferred charges, b) short‐circuit currents, and c) output voltages of DT, PT film, and PT/SiO_2_ film in contact with Cu foil, respectively. d) Influence of SiO_2_ introduced into PT film on adsorption amount of lubricant. e) The effect of different lubricant dosages on the adsorption amount of lubricant for PT/SiO_2_. f) Variation curve of lubricant adsorption amount of PT/SiO_2_ with time. Influence of lubricant volumes on g) short‐circuit currents, h) output voltages of PT/SiO_2_ films. i) Influence of lubricant content on short‐circuit currents of DT–Cu contact. Comparisons of j) transferred charge, k) short‐circuit current, and l) output voltage for 100 and 400 µm thick PT/SiO_2_ films of varying squalane contents. m) Superior long‐term output stability of ERB‐TENG within 35 days. n) Comparison of the output voltage stability between DT‐based interfacial‐lubricating SF‐TENG (DL‐TENG) with 200 µL lubricant and ERB‐TENG under continuous sliding friction for 100 000 cycles. o) Comparison of the device stability of this work with that reported by others.

In the following electrical performance tests, the normal load was set to 5 N at a frequency of 3 Hz, unless otherwise specified. The electric output tests of SF‐TENG were carried out by the measurement platform, shown in Figure S5 (Supporting Information). The research indicated that the electrical output of fabricated PT films diminished, compared to that of DT layers. This decline could be attributed to the porous structure and a significant decrease in effective contact area during the sliding process. Specifically, on the one hand, a decrease in the dielectric constant resulting from the porous structure may lead to a reduction in the electrical output, as presented in **Figures** **3**a–c and S6 (Supporting Information).^[^ ^32^, ^33^, ^34^ ^]^ On the other hand, the porous configuration of the PT films exhibits relatively large pores and features a highly excellent open‐cell structure, thus resulting in high charge dissipation and poor charge storage ability, which causes a further decline in electrical output. Additionally, the surface structure of the PT films displays concave lunar‐like crater structures, which means that during the sliding process, the copper foil cannot come into contact with the sunken part, resulting in a significant reduction in the effective contact area, as well as the electrical output. To address this issue, different ratios of SiO_2_ electret with outstanding charge trapping effect were introduced into PT films for further boosting the dielectric constant and enhancing the electric output.^[^ ^32^ ^]^ The results revealed that the addition of SiO_2_ led to an increase in both output and dielectric constant. However, when the weight ratio of SiO_2_ in THV exceeded 1 wt.%, the output performance slightly decreased due to the decrease in the effective electrification area of THV (Figure S7, Supporting Information). This was attributed to SiO_2_ nanoparticles tending to appear on the surface, as illustrated in Figure S8 (Supporting Information).^[^ ^35^, ^36^ ^]^ As a result, the competition between the increase in charge storage sites and the decrease in the effective THV contact area at high SiO_2_ concentrations led to the final decline in the output of TENGs.^[^ ^35^ ^]^ As an auxiliary proof, the dielectric constant of pristine PT film and PT/ SiO_2_ films with various weight ratios of SiO_2_ exhibited a similar trend as observed in the above‐mentioned output results. The optimal mass ratio of SiO_2_ should be 0.5 wt.%. More importantly, SiO_2_ could be utilized as an oil carriers, lifting the oil absorption of PT/SiO_2_ film by 70.34% in comparison with PT film without SiO_2_ as a result of the large specific surface area of SiO_2_ as well as the presence of numerous active sites on the surface of SiO_2_ that can trap oil (Figure 3d; Note S3, Supporting Information).^[^ ^37^ ^]^

Experiments were conducted to investigate the maximum oil absorption of PT/SiO_2_ films by varying the amount of lubricant. The results manifested that adding 200 µL of squalane reached saturation (Figure 3e). Upon excessive lubricant was added, an excess of lubricant was visibly observed on the surface of the PT/SiO_2_ film (Figure S9, Supporting Information). Furthermore, it was observed that the PT/SiO_2_ film immersed in squalane could reach saturation within 3 min (Figure 3f). Subsequently, the effect of different amounts of lubricant on the electrical output of PT/SiO_2_ films was examined. Results revealed that the addition of low‐content lubricant slightly increased the electrical output while adding 150 µL squalane resulted in a significant boost in output. This improvement can be ascribed to the ability of liquid lubricants to avoid the formation of the transfer film, increase the effective solid–solid contact area, and inhibit interfacial electrostatic breakdown.^[^ ^24^, ^25^ ^]^ Once it reached saturation, there was almost no distinct variation in the output (Figure 3g,h). This phenomenon indicates that there is no need to deliberately control the quantity of oil in PT/SiO_2_ films. In contrast, dense tribo‐layers only showed improved output when 1 µL of lubricant was added, and the electrical output plummeted to a lower level when excessive lubricant was added owing to a reduction in the efficient contact area between tribo‐pairs (Figure 3i; Figure S10, Supporting Information). It can be inferred that it was necessary for dense tribo‐layers to accurately control the lubricant content, and the instability of the lubricant caused by the operational process required periodic replenishment to maintain the output stability (Figure S11, Supporting Information).

It is anticipated that the thickness of PT/SiO_2_ film will impact its maximum oil absorption. Hence, it is imperative to investigate whether an increased film thickness necessitates a higher consumption of lubricating oil. Herein, PT/SiO_2_ film with a thickness of 400 µm is fabricated and examined to evaluate the impact of lubricant content on its output performance. Results exhibit that the output performance is significantly lower than that of the 100 µm thick PT/SiO_2_ film in the absence of lubricant, as illustrated in Figure 3j–l. This discrepancy can be attributed to the weakening of the electrostatic induction to the bottom electrode as the film thickness increases.^[^ ^38^, ^39^ ^]^ The performance of devices with varying film thicknesses provides additional confirmation of this phenomenon (Figure S12a–c, Supporting Information). Interestingly, when adding lubricating oil with the same content as the 100 µm PT/SiO_2_ film (i.e., 200 µL), there is no significant change in output performance for 400 µm PT/SiO_2_ film, but an obvious increase for 100 µm PT/SiO_2_ film. However, it was not until 1000 µL lubricant was added that the output performance of the 400 µm PT/SiO_2_ film significantly improved and tended to stabilize (Figure 3j–l). And more importantly, with only 200 µL of lubricant added, the output voltage of the 400 µm thick PT/SiO_2_ film decreased by 31.9% after 100 000 cycles of durability test (Figure S12d, Supporting Information) and its surface showed noticeable wear (Figure S12e,f, Supporting Information). These results indicate that a positive correlation between film thickness and oil storage capacity. Moreover, a higher volume of lubricating oil is required as the film thickness increases to enhance the lubrication effect, ideally reaching a state of oil absorption saturation.

Since the lubricant self‐replenishing is activated under mechanical stimuli, so it is vital to investigate the influence of the magnitude of the mechanical stimuli on the release of the lubricant and the final output. Specifically, we replaced the Cu foil in ERB‐TENG with cotton sheets of the same size. Alterations were made to the mechanical pressure and following a several‐minute sliding period at 3 Hz, the mass difference of the cotton sheets before and after the test was measured. The volume of lubricant released from the ERB films under different mechanical pressures can be calculated via utilizing Equation 1

(1) $$V=\frac{m}{\rho }$$

where *V* is the volume of lubricant released from the ERB under mechanical pressures; *m* is the mass of the lubricant released from the ERB under mechanical stimuli, which is equivalent to the mass difference of the cotton sheets before and after the test; *ρ* is the density of squalane, *ρ* = 0.81 g ml^−1^. The experimental results indicate that the greater the mechanical stimulation, the more lubricant is released, as illustrated in Figure S13a (Supporting Information). Digital images of the cotton sheets with the fluorescently labeled lubricant absorbed from the ERB film under different mechanical stimulations are shown in Figure S13b,c (Supporting Information). They are illuminated by visible light (Figure S13b, Supporting Information), and an ultraviolet lamp with a wavelength of 365 nm (Figure S13c, Supporting Information), respectively. It is clearly seen that the color intensity of cotton sheets deepens as mechanical stimulation increases, suggesting a higher release of lubricant.

To explore the influence of the magnitude of the mechanical stimuli on the final output, we first investigated the variation in electrical output of PT/SiO_2_ film under various pressures, results revealed that the output initially increased with rising pressure, plateauing at 5 N, then maintaining stability with further pressure increments, as outlined in Figure S13d,e (Supporting Information). For ERB‐TENG, at lower pressures, the electrical output of ERB‐TENG increased with increasing pressures; however, at 7 N pressure, the output declined (Figure S13f,g, Supporting Information), which contrasts sharply with PT/SiO_2_ film without lubricant. This decline could be due to the excessive release of lubricant under high pressures, leading to a decrease in the effective contact area between the two friction materials. This indicates that achieving a controlled release of lubricating oil by regulating external pressure is crucial for ERB‐TENG.

Stability is a vital parameter for TENG devices as it ensures their reliable operation over prolonged durations. Figure S14 (Supporting Information) exhibits the output stability of PT/SiO_2_ over 100 000 cycles. Results indicated the device with the PT/SiO_2_ film showed a 67% drop in output after 9500 cycles. This drop can be ascribed to the destruction of the porous structure of PT/SiO_2_, leading to the formation of the transfer film. Consequently, the transfer film reduced the effective contact area, ultimately causing a decrease in output. Figure 3m showcases that the durability of the ERB‐TENG was retained for up to 35 days, with no significant decline in output performance. Besides, the ERB‐TENG displayed distinguished long‐term output stability, with a decay of only 1% electric output after 100 000 operation cycles, as depicted in Figure 3n. Given the notable impact of lubricant content on the electrical output of DT films, durability tests were performed for 100 000 cycles using both higher lubricant content (200 µL) as well as lower lubricant content (1 µL). The results revealed a sharp decline of 77% and 32% in the electric output of the corresponding SF‐TENG lubricated with 200 µL and 1 µL squalane after 100 000 cycles, respectively (Figure 3n; Figure S15, Supporting Information). In comparison to the state‐of‐the‐art work,^[^ ^15^, ^25^, ^40^, ^41^, ^42^, ^43^, ^44^ ^]^ the stability/decay value reported in this work exceeded the values documented in previous research on ultra‐durable SF‐TENGs (Figure 3o). To further demonstrate the superior performance of the ERB film compared to other materials, we select commonly used electronegative materials like PTFE, FEP, and PI, to compare the electrical output and abrasion resistance of them with the ERB film. The results indicate a significant superiority of the ERB‐TENG in terms of the electrical output (277 V, 2.78 µA, 227 nC), surpassing that of devices utilizing PTFE (107 V, 1.15 µA, 97 nC), FEP (84 V, 1.04 µA, 82 nC), and PI (96 V, 0.80 µA, 70 nC), as illustrated in Figure S16a–c (Supporting Information). Furthermore, the electrical output of the PTFE, FEP, and PI‐based TENG decreased by 45%, 56%, and 77% after 100 000 cycles, respectively, accompanied by noticeable scratches on their surface morphologies, as depicted in Figure S16d and S17 (Supporting Information). These experiments have well verified that ERB has excellent performance compared with other materials. The improved performance of the ERB‐TENG presented in this work provides a potential approach to enhance the output stability and durability of SF‐TENG.

### 2.4 Lubricity and Anti‐Wear Performance of the ERB‐TENG and DL‐TENG

**Figure 4:**
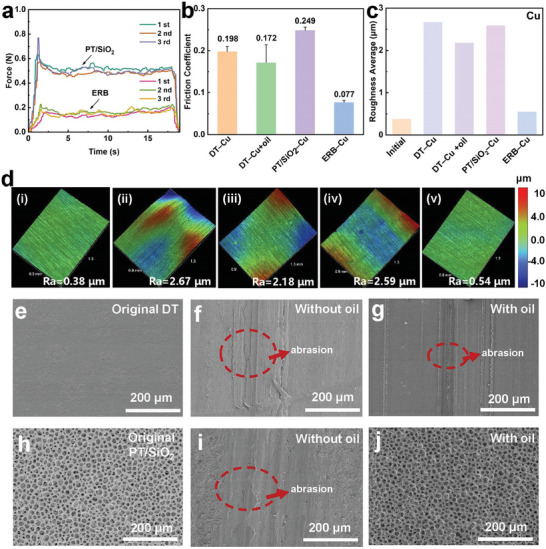
Comparison of the lubricity and long‐term wear resistance between the ERB‐TENG and DL‐TENG with 200 µL lubricant. a) The friction force between PT/SiO_2_ and Cu without oil and with oil. b) The corresponding coefficient of sliding friction of the SF‐TENG with different tribo‐pairs. c) Comparison of the surface roughness of initial Cu foil, as well as Cu foils rubbing with DT and PT/SiO_2_ without oil and in oil after 100 000 sliding friction cycles. d) 3D images were used to depict the surface profile of different Cu foils under varying conditions. These conditions included: i) initial Cu foil, ii) Cu foil in contact with DT without oil, iii) Cu foil in contact with DT with 200 µL oil, iv) Cu foil in contact with PT/SiO_2_ without oil, and v) Cu foil in contact with PT/SiO_2_ with 200 µL oil. 3D images of Cu foils mentioned in ii–v) were tested after 100 000 sliding friction cycles; SEM images of the surface of e) initial DT film, as well as DT films in contact with Cu f) without oil, and g) with 200 µL squalane after continuous 100 000 cycles. h–j) SEM images of the surface of h) initial PT/SiO_2_, and PT/SiO_2_ in contact with Cu i) without oil and j) with 200 µL squalane after 100 000 cycles.

In order to distinctly visualize the surface friction behaviors, a dynamic sliding friction test device was designed (Figure S18, Note S4, Supporting Information). The friction force and corresponding coefficient of sliding friction (COF) were tested for each material pair mentioned above, following the GB 10006‐88 standard. Three measurements were taken to obtain experimental results. The addition of squalane to PT/SiO_2_‐Cu tribo‐pair resulted in a significant reduction of 69% for the friction force and COF, as depicted in **Figure** **4**a,b. In contrast, when oil was added to the contact interface of DT‐Cu tribo‐pair, the friction force and COF decreased by only 13% (Figure S19, Supporting Information; Figure 4b). This phenomenon can be explained by two factors. On the one hand, the ERB film with the self‐replenishment property releases the lubricant, distributing it evenly into small droplets to achieve interfacial lubrication in the ERB‐Cu tribo‐pair. On the other hand, when a lubricant is applied to the contact interface of the smooth DT film and the Cu foil, a continuous oil film with a specific thickness is formed, expelling air from the interface. Subsequently, the atmospheric pressure acts, causing an increase in the friction force between the two interfaces. This hinders the lubricating effect of the lubricant, resulting in only a slight decrease in both the friction force and COF. For further evaluating the wear resistance of the self‐replenishing lubricating ERB film, the surface roughness of Cu foils and corresponding SEM images of various tribo‐layers were analyzed before and after 100 000 cycles of sliding. The results showed that the initial dense Cu foil features a smooth surface (Figure S20a, Supporting Information) with a surface roughness of only 0.38 µm (Figure 4c,d‐i)). Nevertheless, in the case of the DT‐Cu tribo‐pair, the addition of lubricant (1 or 200 µL) could not prevent substantial wear on the Cu surface (Figures S20 and S21, Supporting Information). Even when 200 µL of lubricant was applied, the surface roughness of the Cu demonstrated a substantial increase, more than 4.7 times compared to that of the pristine Cu sample (Figure 4c,d‐iii)). It is noteworthy that for the PT/SiO_2_‐Cu tribo‐pair, same as the DT‐Cu tribo‐pair, significant wear was observed on the Cu surface in the absence of any lubricant (Figure 4d‐iv; Figure S22a, Supporting Information). However, upon the addition of 200 µL of lubricant, the Cu surface exhibited virtually no discernible wear (Figure S22b, Supporting Information), with the surface roughness increasing by a mere 42% as compared to the untreated Cu sample (Figure 4c, d‐v)).

The wear characteristics of the DT and PT/SiO_2_ tribo‐layers which were in contact with Cu foils demonstrated wear patterns that paralleled the trends observed in the wear of the Cu surface, as previously mentioned. Specifically, the original DT film exhibited a highly smooth surface (Figure 4e). However, significant scratching was evident after 100 000 sliding friction cycles (Figure 4f), with substantial wear still observable despite the application of either 1 or 200 µL of lubricant (Figure S23, Supporting Information; Figure 4g). This finding is consistent with its high friction resistance and COF. For the PT/SiO_2_ film, the surface experienced severe degradation in the absence of lubricant, and even its porous structure was damaged (Figure 4h,i). Yet, post‐treatment with 200 µL of lubricant (ERB), the surface morphology remained nearly unchanged, indicating remarkable wear resistance (Figure 4j). This enhanced durability can be ascribed to the lubricant being harbored within the porous structure, endowing the film with self‐lubricating properties that significantly reduce the COF and consequently play a role in friction reduction and wear resistance enhancement.

### 2.5 Application Demonstrations of ERB‐TENG

**Figure 5:**
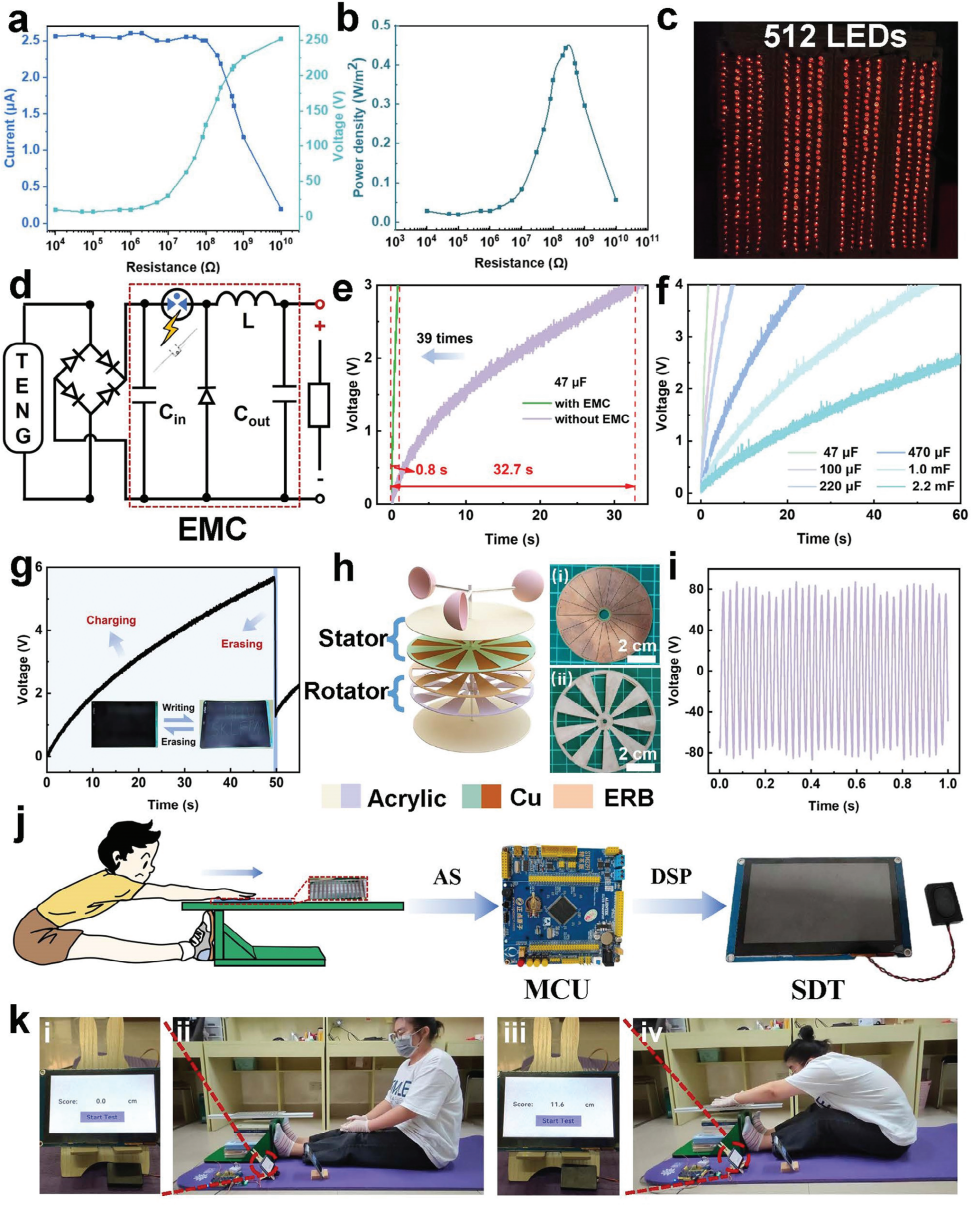
Charging performance characterizations and application demonstrations of the ERB‐TENG. a) Instantaneous peak V_oc_ and I_sc_ of ERB‐TENG concerning load resistance. b) Power density of the ERB‐TENG concerning a load resistance of 10^4^–10^10^ Ω. c) Photograph of 512 LEDs lighted instantaneously by arrayed ERB‐TENG. d) Equivalent circuit diagram of the integrated energy management circuit (EMC) for the arrayed ERB‐TENG. e) Comparison diagram of the charging speed of 47 µF capacitor by the arrayed ERB‐TENG with and without EMC. f) Charging curve of different capacitors by the arrayed ERB‐TENG with EMC. g) Charging curve of a 470 µF commercial capacitor upon powering a 20‐inch color LCD blackboard driven by arrayed ERB‐TENG. Inset: enlarged view of the discharge curve when erasing the handwriting on the LCD blackboard. h) Schematic view of the structure and materials for the electrode stator and the ERB rotator. Photographs of the electrode stator: i) and the ERB rotator ii). i) The V_oc_ of the ER‐TENG was obtained at the rotating speed of 270 rpm by harvesting wind energy. j) Signal flowchart of the “sit and reach” intelligence test. k) Demonstrations of the “sit and reach” test system with ERB‐TENG for intelligent sports monitoring. Photographs of initial state (i and ii) and status after measurement (iii and iv).

As a kind of device that converts low‐quality environmental energy into electricity, TENGs have been extensively applied in many scenarios. The output power of a TENG under practical conditions depends on its load resistance. **Figure** **5**a,b systematically investigated the output voltage, current, and power density of the ERB‐TENG dependent on external load resistances from 10^4^ to 10^10^ Ω. The instantaneous power density on the load reaches a maximum value of 0.45 W m^−2^ at the matched resistance of 304 MΩ. An arrayed ERB‐TENG consisting of two sections is developed for driving commercial electronics and demonstrating the charging performance: an ERB film attached to 16 staggered electrodes as a slider, and 8 separate Cu foils adhered to the substrate as a stator. Figure 5c exhibits the arrayed ERB‐TENG directly illuminating 512 LEDs (Figure S24 and Movie S1, Supporting Information). An energy management circuit (EMC) is designed that is aimed at addressing the issue of low charging efficiency resulting from circuit loss during the practical application of SF‐TENGs, depicted in Figure 5d. The specific working mechanism is briefly described below. Here, a matching capacitance C_in_ is introduced to store the unstable energy generated by the SF‐TENG via full‐wave rectification, and a gas discharge tube (GDT) is adopted as a switch to efficiently release accumulated energy. The GDT is off till the voltage of C_in_ exceeds the threshold voltage of the discharge tube, allowing the instantaneous release of energy from C_in_ to the inductor via gas breakdown discharge. Subsequently, the energy in the inductor will continuously power the capacitor C_out_ through a closed‐loop circuit, significantly improving the charging speed. Specifically, the arrayed ERB‐TENG is combined with the EMC to charge a 47 µF capacitor, it is able to charge the voltage to 3 V in 0.8 s. However, without the EMC, it takes 32.7 s to charge the capacitor to the voltage of 3 V. This result indicated that the introduction of EMC can remarkably boost the charging efficiency by 39 times, as illustrated in Figure 5e. The charging performance of the device with EMC at various capacitances (47, 100, 220, 470 µF, 1, 2.2 mF) is also examined, as presented in Figure 5f. The voltage on the capacitor of 220 µF can be charged to 4 V within 8 s, highlighting its excellent practical feasibility for driving multiple commercial electronics. Figure 5g shows the charge‐discharge curve of the 20‐inch LCD blackboard recorded during the whole operation, enabling handwriting and erasure functions (Movie S2, Supporting Information). The ERB‐TENG can be further developed into a rotary‐freestanding TENG (ER‐TENG), which is desirable for more application scenarios, such as efficiently harnessing wind energy. The basic structure of the ER‐TENG is illustrated in Figure 5h, which consists of a rotator, a stator, a wind cup, and a shell. The breeze from the blower enables the output voltage, current, and transferred charge of the ER‐TENG to reach 90 V, 1.25 µA, and 11 nC at a rotation speed of 270 rpm, respectively, as outlined in Figure 5i; Figure S25 and Movie S3 (Supporting Information). The ER‐TENG mentioned above offers significant advantages for wind energy harvesting, presenting a promising development prospect.

Nowadays, smart sports facilities and wearable equipment are gaining popularity in the realm of sports, as it undergoes a digital and intellectual transformation.^[^ ^45^ ^]^ The “sit and reach” test is a mandatory assessment in the “National Student Physical Fitness and Health Standards” to evaluate the flexibility of adolescents' bodies. Here we designed a self‐powered “sit and reach” system, which can provide real‐time vocal announcements of the test results. This system introduces a single‐electrode arrayed ERB‐TENG as a sensor which consists of two components: a slider and a stator. For the stator, the ERB is securely attached to 14 conductive fabrics. As for the slider, the electropositive material is affixed to the right index finger of the tester. As the finger slides over the stator, continuous pulse electrical signals are generated, consistent with the number of conductive fabric electrodes contacted by the finger, serving as analog signals (AS). AS are subsequently transmitted to a microcontroller unit (MCU) to be converted into digital signals (DS) and undergo further processing. The automatic multi‐scale peak detection algorithm is employed to automatically detect the number of digital signal peaks (DSP), which is utilized to calculate the finger sliding distance (*d*) by the following Equation 2:

(2) $$d=n*d_{0}+\left(n-1\right)*d_{1}$$

where *d* is the grade of a conner in sit and reach too, *n* represents the number of signal peaks, *d*_0_ is the width of each conductive fabric, and *d*_1_ is the width of the intermediate gap between adjacent conductive fabrics. Subsequently, MCU transmits *d* to the universal asynchronous receiver‐transmitter (UART), thus a screen display terminal (SDT) receives the data via specific protocols in UART and presents it on the SDT. Furthermore, the voice synthesis module in SDT synthesizes the prompt message and broadcasts it via the speaker. The corresponding signal flowchart as well as application illustration are illustrated in Figure 5j,k (Movie S4, Supporting Information). The system possessing a great merit of durability can realize smarter and more convenient detection for the “sit and reach” test.

## 3 Conclusion

In conclusion, we have successfully developed an ultra‐durable and stable SF‐TENG utilizing an ERB film as a tribo‐material. This bionic film is constructed by incorporating lubricant within a 3D porous structure, demonstrating remarkable output stability and outstanding wear resistance. It can continually lubricate the friction interface when subjected to mechanical stimulation, thereby addressing the issue of precise control and periodic replenishment of lubricant content in DT films. Additionally, the ERB film with a COF of 0.077 demonstrates outstanding performance with no discernible surface wear after 100 000 sliding friction cycles. Furthermore, it only experiences a minimal 1% decrease in electrical output. This real‐time replenishment mechanism ensures a consistent output and extends the service life of the SF‐TENGs. Thus, this work presents a promising approach to enhance the durability and output stability of SF‐TENG devices, which have been applied to energy harvesting and intelligent sports monitoring.

## 4 Experimental Section

### Materials

3 M Dyneon Fluoroplastic THV 221 AZ and PET film were obtained from Minnesota Mining and Manufacturing Co., Ltd. O‐SiO_2_ with a diameter of 15 ± 5 nm were supplied by Shanghai Macklin Biochemical Co., Ltd. DMF and acetone were provided by Sinopharm Chemical Reagent Co., Ltd. Squalane was purchased from Shanghai Aladdin Biochemical Technology Co., Ltd. Other commercial materials, including dense PTFE (100 µm thick), dense FEP (100 µm thick), dense PI (100 µm thick), foam tape, polymethyl methacrylate (PMMA) substrate, Cu foils, and conductive fabric tape (SY‐NF35D‐C, PET/Cu + Ni) were purchased by a local distributor.

### Preparation of the PT Film, PT/SiO_2_ Film, ERB Film, and DT Film

To prepare the PT film, THV particles (4.5 g) were added to a mixture of DMF (8.5 g) and acetone (17 g), then heated on a magnetic stirrer at 100 °C for 5 h to obtain a homogeneous solution. The mixed solution was then poured onto a substrate and blade‐coated using a coating machine (AFA‐IV, Xiandaihuanjing, China). Next, it was transferred to a constant temperature and humidity chamber (HWS‐80F, Shanghai Zhengqiao Scientific Instrument Co., Ltd) with a humidity of 40% to produce a PT film with a thickness of 100 µm. For preparing PT/SiO_2_ film, SiO_2_ (0.0225 g) was added to the mixed solution with DMF (8.5 g) and acetone (17 g), then dispersed evenly under ultrasonication for 1 h. The following part is the same as the steps above for preparing PT film to obtain PT/SiO_2_ film. Then, the fabricated PT/SiO_2_ was immersed in squalane until it reached the saturation point of the oil to obtain the ERB film. Analogously, the THV particles were dissolved in DMF at 100 °C for 5 h, then transferred to an oven curing at 80 °C for 3 h for fabricating the DT film. The thickness of the electronegative materials involved in this work is 100 µm.

### Fabrication of the ERB‐TENG, the Arrayed ERB‐TENG, and the Single‐Electrode Arrayed ERB‐TENG

For the ERB‐TENG, two pieces of acrylic sheets with a thickness of 3 mm were cut by a laser cutter (GCC Laser Pro) as substrates. For the stator, one of the acrylic sheets was cut into a square shape with the dimension of 30 × 30 mm^2^, a piece of foam tape of the same size was attached to this acrylic sheet to ensure contact intimacy, and then Cu foil was adhered to the foam tape. For the slider, two pieces of conductive fabrics (30 × 30 mm^2^) with a small gap of 1 mm were adhered to the acrylic sheet (100 × 60 mm^2^), acting as the complementary electrodes, then the ERB film (100 µm thick) was adhered to the conductive fabrics. In the case of the arrayed ERB‐TENG, for the slider, 16 conductive fabrics with a gap of 1 mm that adhered to an acrylic sheet, were utilized as interdigital electrodes, the size of each one was 45 × 11 mm^2^, thus ERB was attached to the entire surface of conductive fabrics, acting as a negative material. For the stator, 8 Cu foil arrays with a gap of 1 mm were attached to the other acrylic sheet, serving as the positive materials. The single‐electrode arrayed ERB‐TENG consists of two components: a slider and a stator. For the stator, the ERB was securely attached to 14 conductive fabrics, each measuring 80 mm in length and 8 mm in width, spaced equidistant at intervals of 10 mm. As for the slider, the electropositive material of the same dimension was affixed to the right index finger.

### Fabrication of the DL‐TENG

For a stator, a piece of foam tape was attached to the acrylic substrates (30 × 30 × 3 mm^3^) to improve the contact efficiency, and then the Cu foil with the same size was adhered to the foam, as a positive material. For the slider, DT film with a thickness of 100 µm was adhered to the conductive fabrics, acting as a negative material, which in turn were adhered to the other acrylic sheet (100 × 60 × 3 mm^3^). The size of the foam and conductive fabrics were all 30 × 30 mm^2^. In the lubrication tests, different liquid lubricant content was added to the triboelectric interface.

### Fabrication of the ER‐TENG

The prepared ER‐TENG was composed of four parts, namely the wind cup, shell, electrode stator, and the ERB rotator. The commercial wind cup had a diameter of 220 mm and a height of 110 mm. The metal rotator was composed of radially arranged sectors with equal intervals. The shell had inner and outer diameters of 80 and 130 mm, respectively, with a height of 40 mm. For the electrode stator, the inner and outer diameters of the epoxy glass cloth laminate sheet substrate (FR‐4) (1.6 mm thick) were 7 and 70 mm, respectively. It was fixed beneath the top acrylic sheet. The Cu layers (35 µm thick) with two groups of complementary radial‐arrayed sectors (a total of 18 sectors, 0.3 mm gap distance) with the same central angle were coated on the substrate utilizing printed circuit board techniques. The inner and outer diameters of the Cu electrode were 11.5 and 69 mm, respectively. Vias were drilled at the edge of the Cu electrode to connect the wires from the other side. For the ERB rotator, the ERB layer (100 µm thick) adhered to the acrylic substrate (4 mm thick) serving as the negative triboelectric material. A radial‐arrayed structure with nine evenly arranged hollowed‐out sectors (central angle ≈20°) with an outer diameter of 70 mm and an inner diameter of 2 mm was shaped using a laser cutter.

### Measurement of the COF

For the dynamic sliding friction test, the THV film was coated on the glass plate fixed on an optical platform, and the Cu foil with the dimension of 63 × 63 mm^2^ was attached to the lower surface of an iron block (200 g) with the same size. The iron block was placed on the THV film and one side was attached to the hook of the digital force gauge (SH‐5, rated load 5 N, HANDPI, China) to record the variation in the friction force. In addition, the gauge was fixed on an acrylic cantilever beam connected to a linear motor (R‐LP3, China), which controlled the uniform sliding velocity (150± 10 mm min^−1^) and distance (30 mm) of the iron block.

### Characterization

The optical images of the earthworm were taken with an ultra‐depth field microscope (DVM6, Leica, Germany). The 3D images and roughness of contact surfaces were investigated via an optical profiler (Ni9100, Veeco, USA). FE‐SEM (SU8010, Hitachi, Japan) was used to observe the morphologies and microstructures of tribo‐layers. The output measurements of SF‐TENGs were carried out under the reciprocating sliding motion produced by a custom‐made linear motor. The output electric signals were measured by using an electrometer (Keithley 6514, USA) and an Oscilloscope (Wavesurfer 104MXs‐B, LeCroy, USA) with a probe impedance of 100 MΩ.

## Conflict of Interest

The authors declare no conflict of interest.

## Supporting information

Supporting Information

Supplemental Movie 1

Supplemental Movie 2

Supplemental Movie 3

Supplemental Movie 4

## Data Availability

The data that support the findings of this study are available from the corresponding author upon reasonable request.
