# Supplementary material for: Earthworm‐Inspired Ultra‐Durable Sliding Triboelectric Nanogenerator with Bionic Self‐Replenishing Lubricating Property for Wind Energy Harvesting and Self‐Powered Intelligent Sports Monitoring
Source: Adv Sci (Weinh). 2024 May 13;11(28):2401636. doi: 10.1002/advs.202401636 (PMC11267296; doi:10.1002/advs.202401636)
Supplement: Supplementary file 1 — Supporting Information [file ADVS-11-2401636-s002.docx]

# Earthworm-Inspired Ultra-Durable Sliding Triboelectric Nanogenerator with Bionic Self-Replenishing Lubricating Property for Wind Energy Harvesting and Self-Powered Intelligent Sports Monitoring

*Mengjiao Liu, Xin Zhang, Yue Xin, Dongxu Guo, Guangkai Hu, Yifei Ma, Bin Yu*, Tao Huang*, Chengchang Ji*, Meifang Zhu, Hao Yu*

M. Liu, X. Zhang, G. Hu, B. Yu, T. Huang, C. Ji, M. Zhu, H. Yu

State Key Laboratory for Modification of Chemical Fibers and Polymer Materials

College of Materials Science and Engineering

Donghua University

Shanghai 201620, China
E-mail: [yubin@dhu.edu.cn](mailto:yubin@dhu.edu.cn); ht@dhu.edu.cn; [jicc@dhu.edu.cn](mailto:jicc@dhu.edu.cn);

Y. Xin, Y. Ma

College of Information Science and Technology

Donghua University

Shanghai 201620, China

D. Guo

College of Computer Science and Technology

Donghua University

Shanghai 201620, China

G. Hu

Lehrstuhl für Chemische Reaktionstechnik

Friedrich-Alexander-Universität Erlangen-Nürnberg

Erlangen, 91058, Germany

**Supplementary Notes**

**Note S1. Porous structure formation process of porous THV films**

The formation of both internal and external porous structures in polymer thin films can be ascribed to the synergetic effect of solvent evaporation and relative humidity.^[1]^ In order to gain a comprehensive understanding of the pore formation mechanism, two models for phase separation,^[1-5]^ namely, EIPS and VIPS, have been proposed to account for the formation of microscale pores in polymer thin films. In EIPS, phase separation occurs due to the rapid evaporation of the solvent, leading to a decrease in temperature and causing the solution to pass through the bimodal curve of a phase diagram for entering the metastable region. This transition induces phase separation into polymer-rich and solvent-rich regions. Subsequently, the polymer-rich phase will then transform into a solid matrix while the solvent-rich phase will evolve into pores after the solvent has evaporated. The EIPS plays an essential role in the surface pore formation of the porous THV film. The other model, VIPS model proposes that the penetration of a nonsolvent (water vapor) into the polymer solution triggers a phase separation of the polymer, resulting in the generation of inherent porous structure in the polymer. The nonsolvent causes the polymer to precipitate out of the solution, forming the solid matrix, while the solvent-rich phase evolves into porous regions.

**Note S2. Porosity of porous THV films**

The dimension of all films involved was 3 × 3 × 0.01 cm^3^. The porosity (***P***) was tested by weight measurement method, which could be obtained by the equation:

$\text{P }\left( \text{\%} \right)\text{ = }\frac{\text{(}\text{V}_{\text{1 }}\text{- }\text{V}_{\text{0}}\text{)}}{\text{ }\text{V}_{\text{1}}}\text{ = }\frac{\left( \text{V}_{\text{1}}\text{-}\frac{\text{m}_{\text{0}}}{\text{ρ}_{\text{0}}} \right)}{\text{V}_{\text{1}}}\text{ }\text{=}\text{ }\frac{\left( \text{V}_{\text{1}}\text{-}\frac{\text{m}_{\text{0}}}{\text{ρ}_{\text{2}}} \right)}{\text{V}_{\text{1}}}$ (1) $\text{ρ}_{\text{0}}\text{ }\text{=}{\text{ }\text{ρ}}_{\text{2}}\text{ }\text{=}\text{ }\frac{\text{m}_{\text{2}}}{\text{V}_{\text{2}}}$ (2)

*V_0_*, $\text{m}_{\text{0}}$, $\text{ρ}_{\text{0}}$ — were the skeleton volume, mass and density of the porous THV material.

*V_1_* — The volume of a porous THV material in its natural state.

*V_2_*, $\text{m}_{\text{2}}$, $\text{ρ}_{\text{2}}$ — were the absolute compact volume, mass and density of the dense THV material.

**Note S3. Lubricant Adsorption**

Porous THV samples (*m_0_*) were immersed in lubricant for a period of time. Then, one end was clamped with tweezers to separate the sample from the liquid until the lubricant was no longer dripping. Subsequently, the samples were weighed (*m_1_*). The lubricant adsorption of porous THV films is calculated by the following formula:

$\text{Weight gain (}\text{\%}\text{) = }\frac{\left( \text{m}_{\text{1}}\text{-}\left. \text{m}_{\text{0}} \right) \right.}{\text{m}_{\text{0}}}$ (3)

**Note S4. The COF calculation in the dynamic sliding friction test**

Schematic illustration of the dynamic sliding friction test result for different triboelectric material pairs is shown in Figure 4b. According to the force balance condition, the measurement result of the commercial digital force gauge is the friction force of the tribo-pair when the iron block slides with a constant speed. The dynamic COF of film samples obtained via the dynamic sliding friction test can be calculated by the following equations:

$\text{μ}_{\text{d}}\text{=}\frac{\text{F}_{\text{d}}}{\text{F}_{\text{N}}}$ (4)

where *F_d_* is the average dynamic friction force in the process of the uniform sliding, *F_N_* is the normal force on the sliding iron block, *μ*_d_ is the dynamic COF obtained via the dynamic sliding friction test. The arithmetic mean of COFs is calculated by three test results.

**Supplementary Figures**


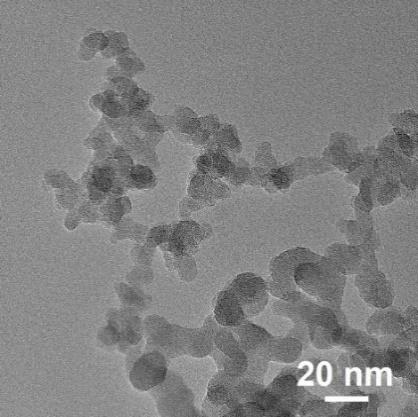


**Figure S1.** Image of transmission electron microscopy of SiO_2_ with particle size of 15 nm ± 5 nm.

**
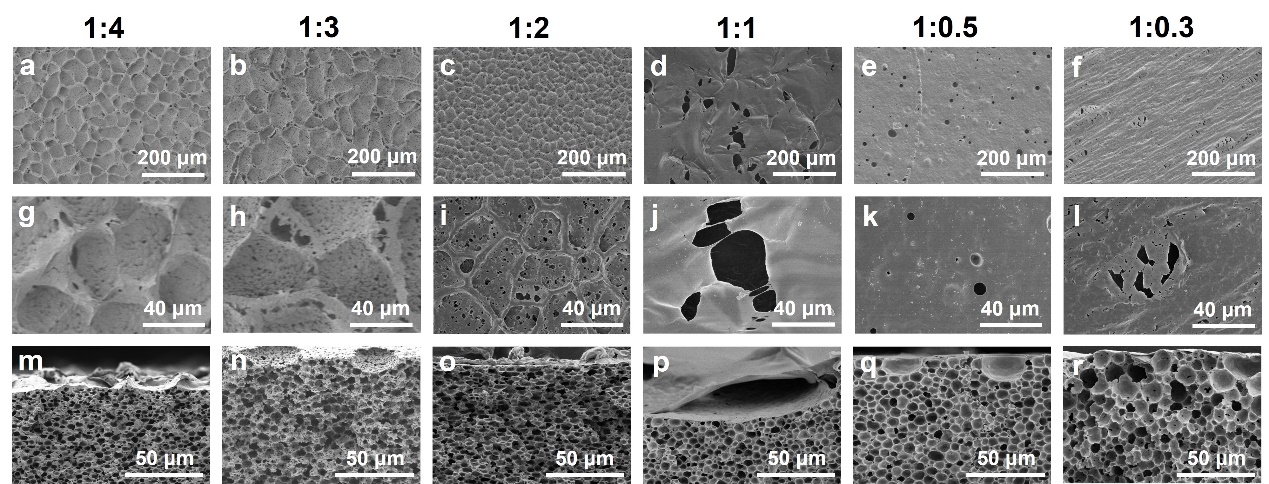
**

**Figure S2.** Representative surface, corresponding surface enlarged view and cross section scanning electron microscopy images of PT samples fabricated by 15 wt% THV solutions from DMF/acetone with different solvent ratios (1:4, 1:3, 1:2, 1:1, 1:0.5, 1:0.3) under the relative humidity of 55% ± 3% via EIPS and VIPS are shown in a-f, g-l and m-r, respectively.

**
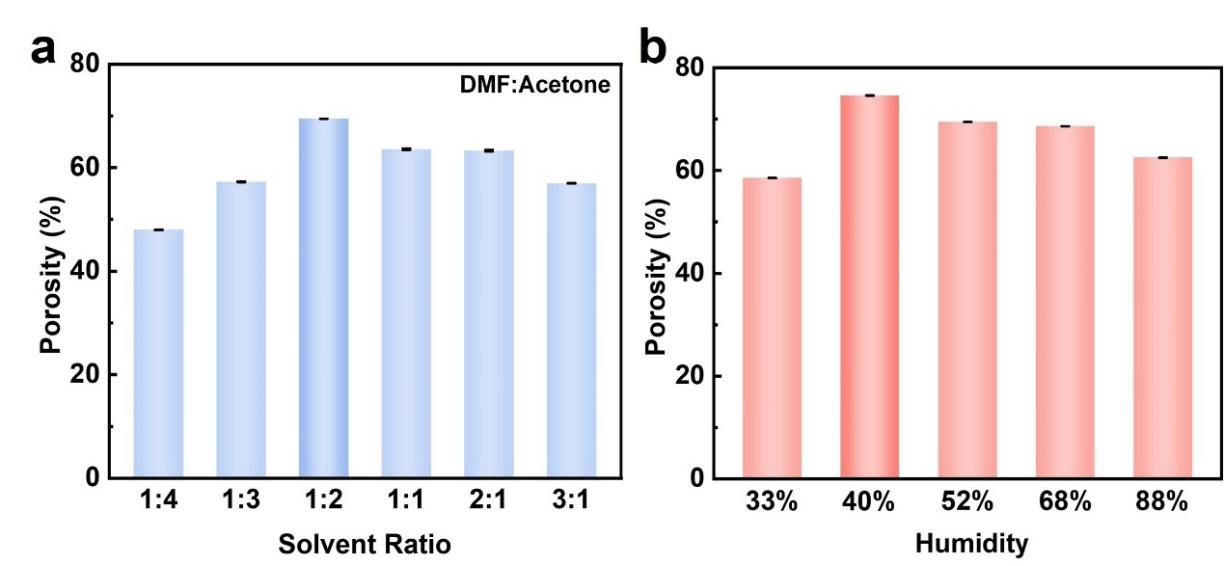
**

**Figure S3.** a) Porosity of PT samples fabricated by 15 wt% THV solutions from DMF/acetone with different solvent ratios (1:4, 1:3, 1:2, 1:1, 2:1, 3:1) under the relative humidity of 55% ± 3%. b) Comparison of the porosity of PT films fabricated by a 15 wt% THV solution in DMF/acetone (1:2, w/w) under different relative humidity (33%, 40%, 52%, 68%, 88%).


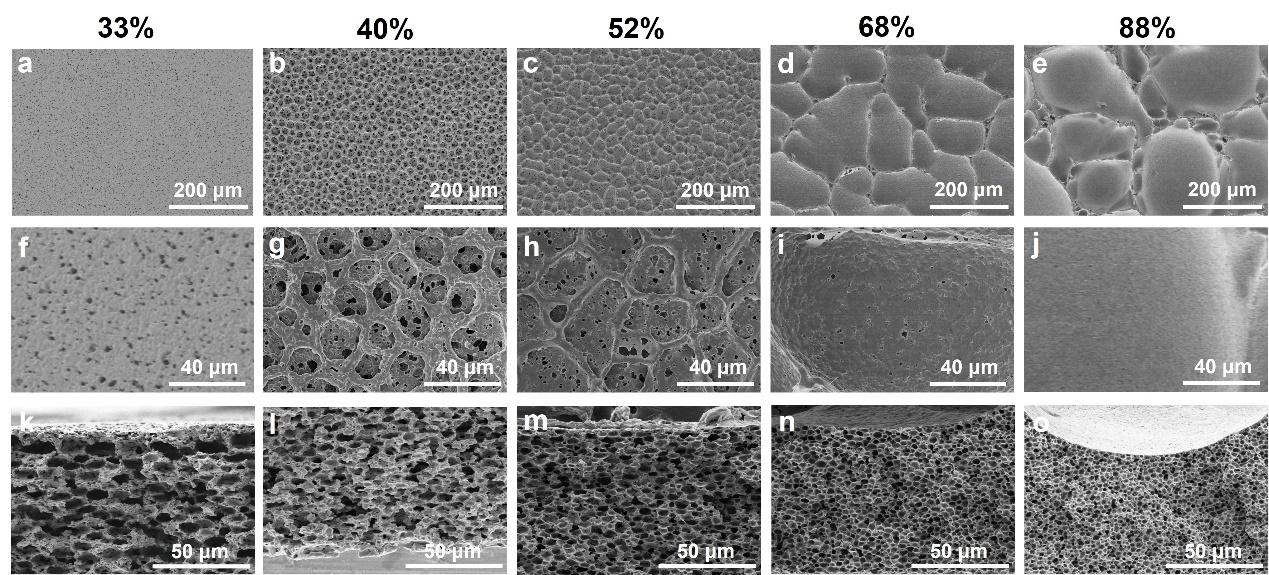


**Figure S4.** Comparison of (a-e) the surfaces, (f-j) corresponding surface enlarged view and (k-o) cross sections SEM images of PT films fabricated by a 15 wt% THV solution in DMF/acetone (1:2, w/w) under different relative humidity: (a, f, k) 33%, (b, g, l) 40%, (c, h, m) 52%, (d, i, n) 68%, (e, j, o) 88%. The porous structure of samples is prepared by EIPS and VIPS. Humidity error range: ± 3%.


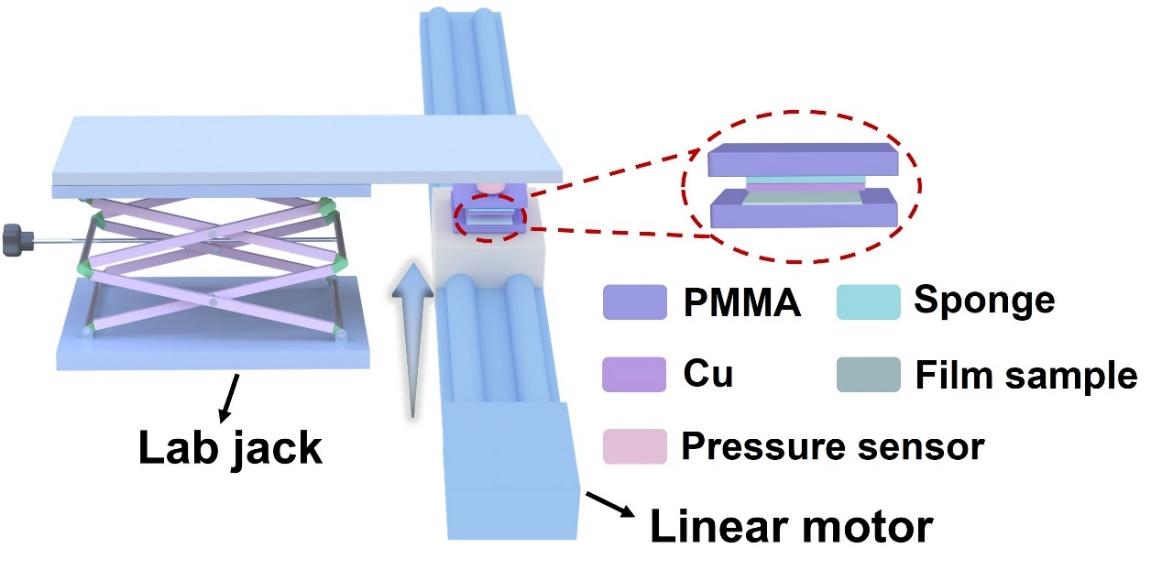


**Figure S5.** Schematic diagram of the electrical output measurement platform. The inset is an enlarged view of the measurement part of the device on the platform.


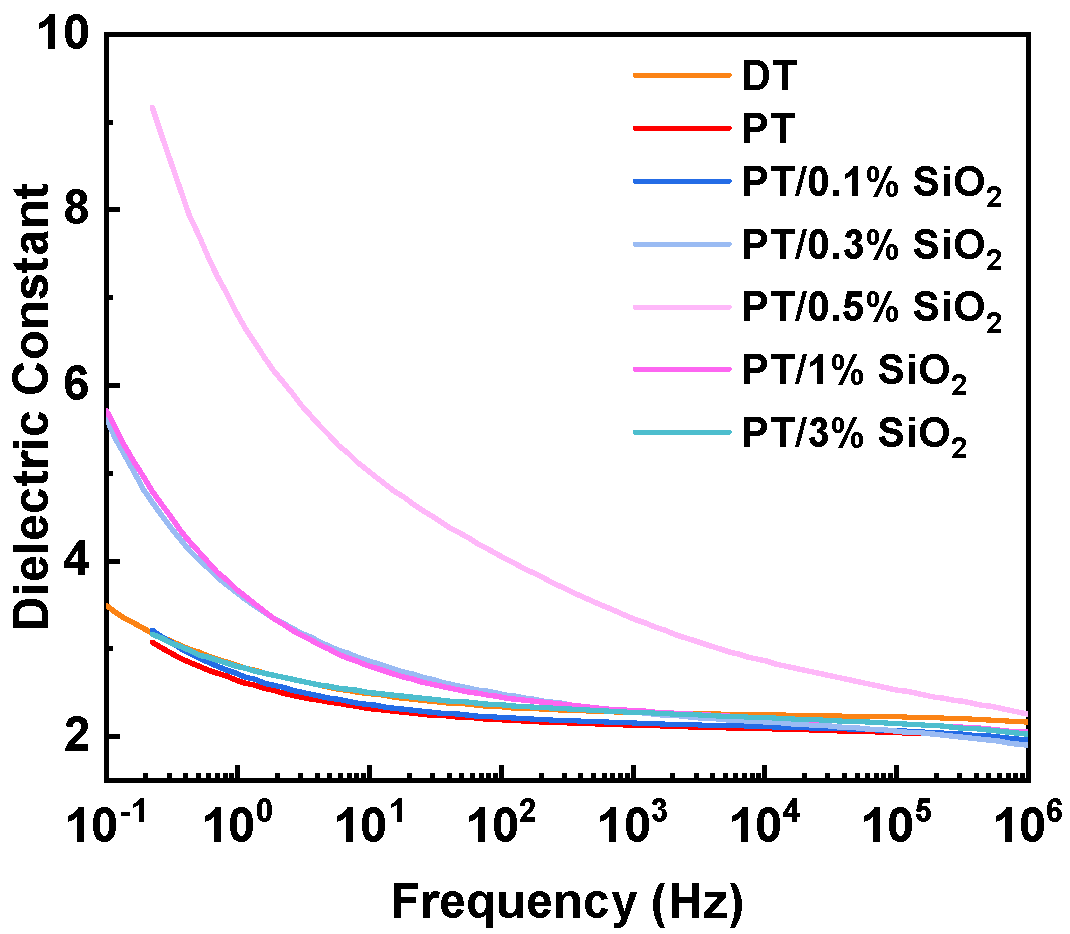


**Figure S6.** Dielectric constant of DT, PT and PT doped with different mass ratios of SiO_2_.


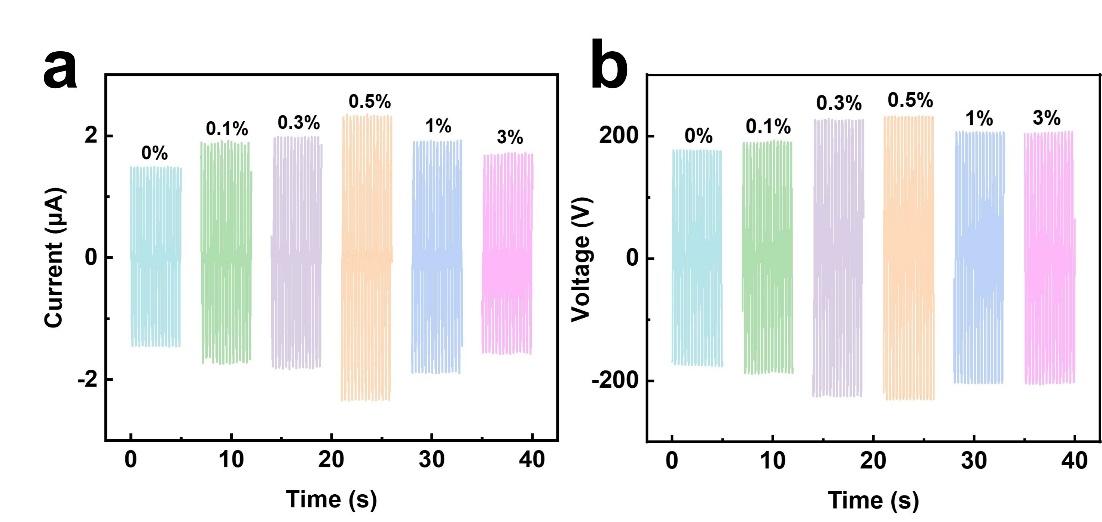


**Figure S7.** Influence of different mass ratios of SiO_2_ on (a) short-circuit current and (b) open-circuit voltage of PT/ SiO_2_-Cu tribo-pair.


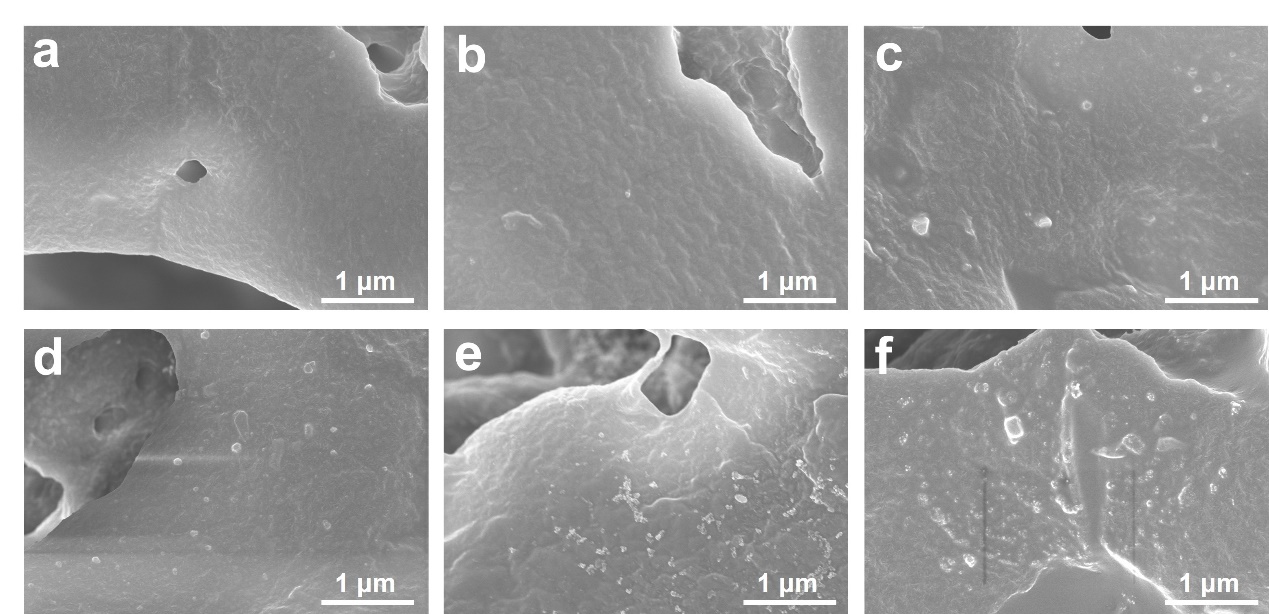


**Figure S8.** SEM images of the surface of PT/SiO_2_ with the SiO_2_ concentration of (a) 0, (b) 0.1, (c) 0.3, (d) 0.5, (e) 1, (f) 3 wt.%.


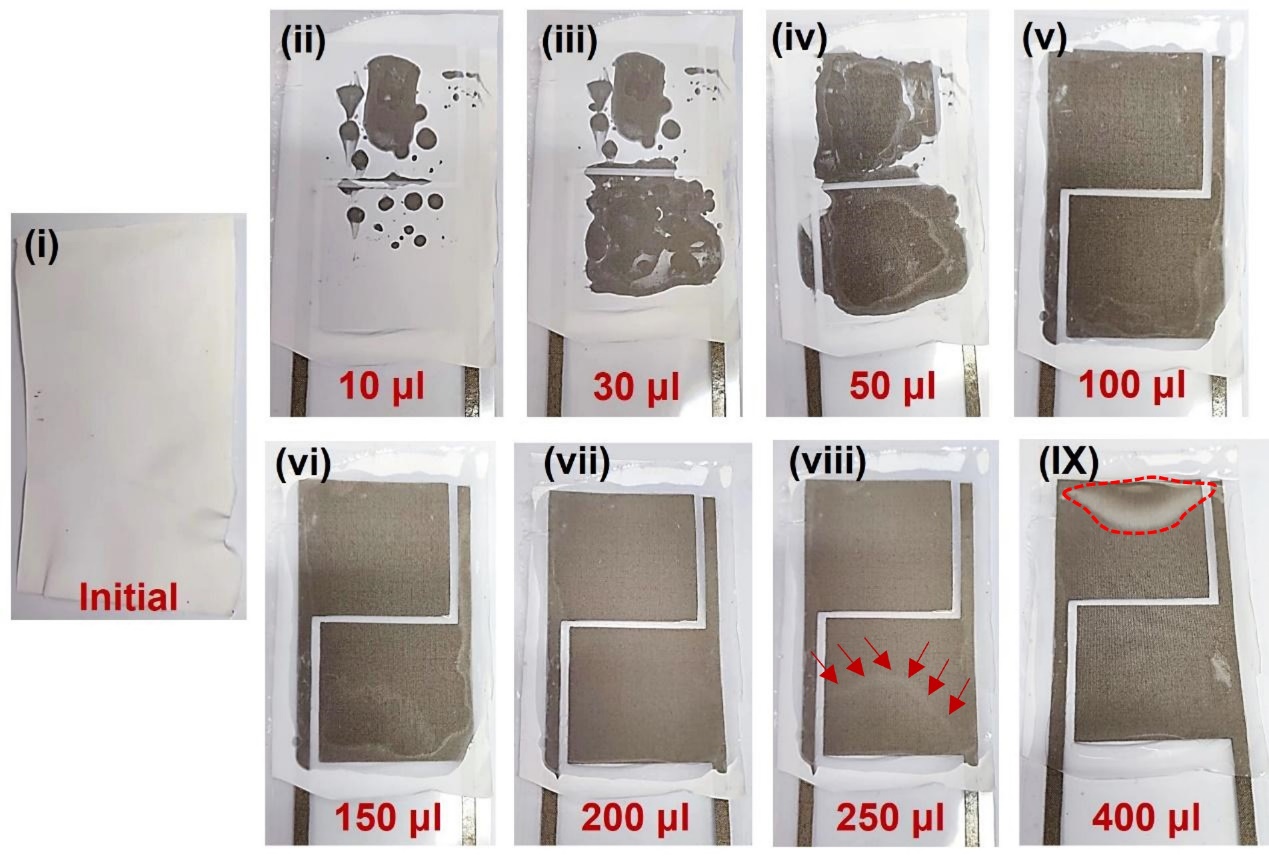


**Figure S9.** Digital images of PT/SiO_2_ with different squalane volumes.


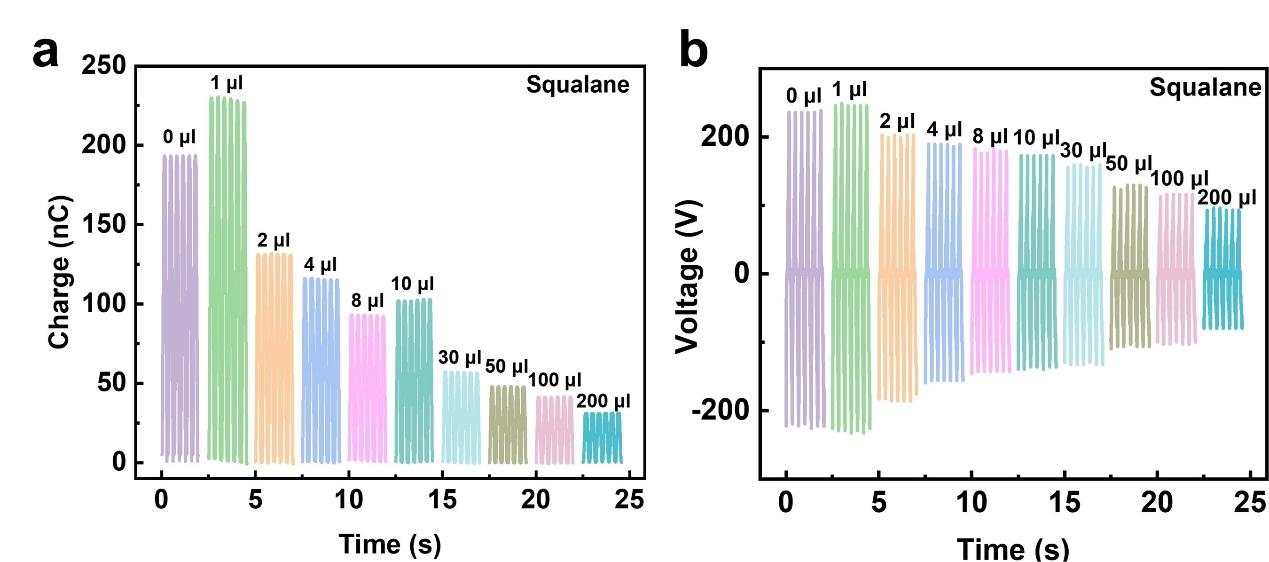


**Figure S10.** Influence of different lubricant volumes on output of DT–Cu contacts. a) transferred charges, b) open-circuit voltage.


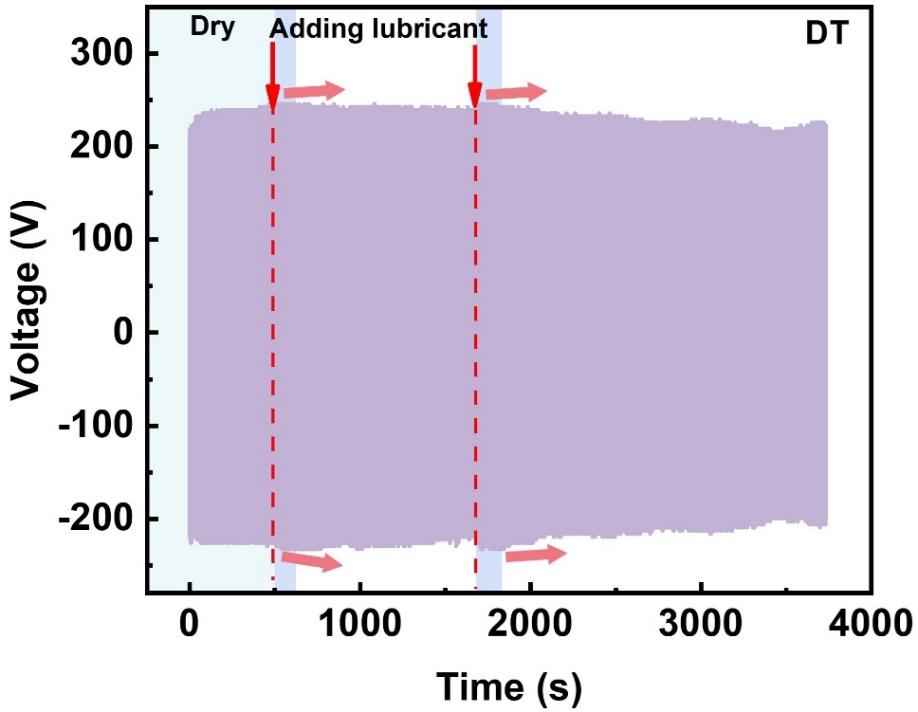


**Figure S11.** Influence of in situ adding 1 μL squalane to DT–Cu contact on output.


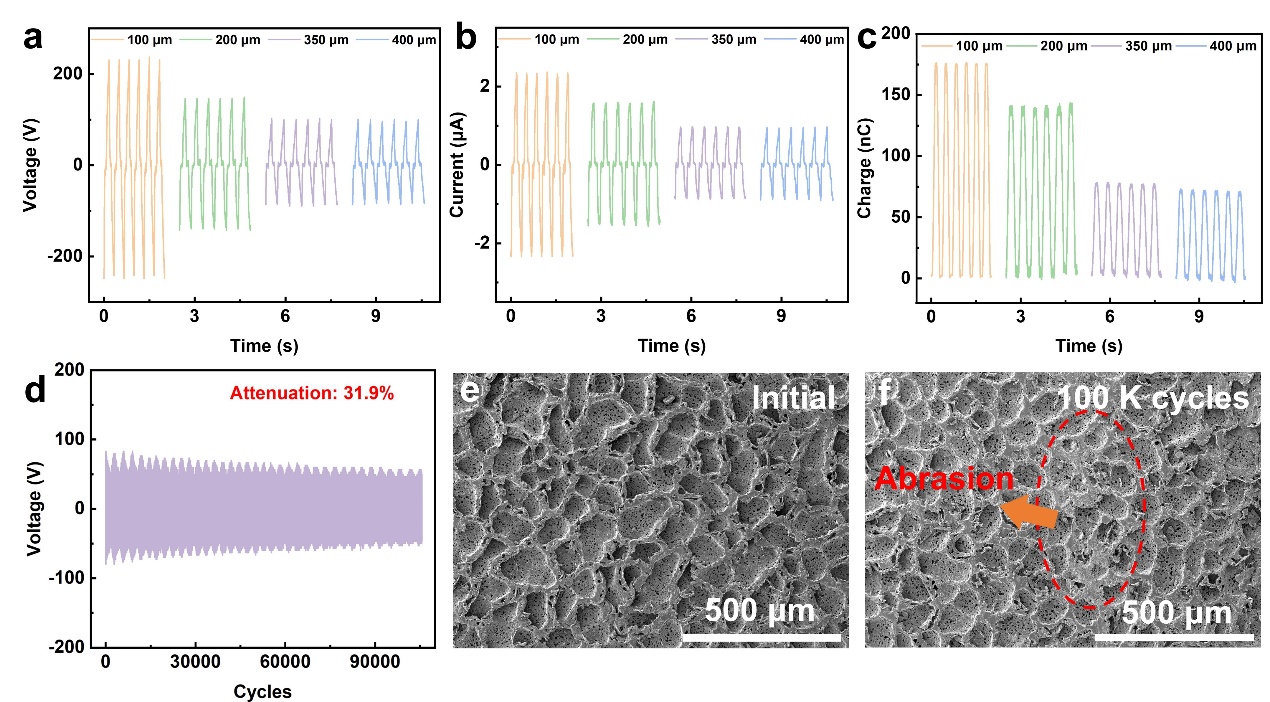


**Figure S12**. The comparisons of (a) output voltage, (b) short-circuit current, (c) transferred charge of PT/SiO_2_ with different thicknesses. d) The output stability and SEM images of surface morphology of 400 μm thick PT/SiO_2_ with 200 μL squalane (e) before and (f) after 100, 000 cycles.


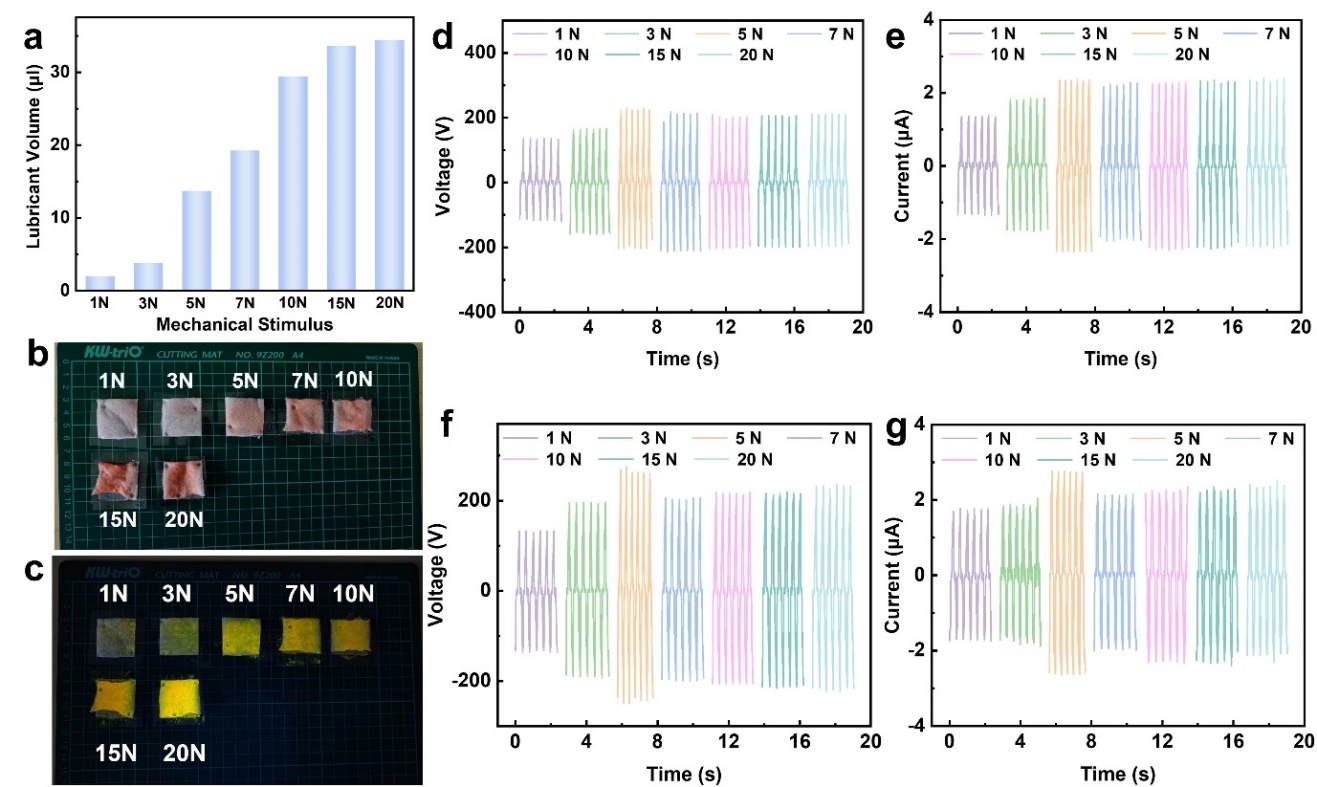


**Figure S13.** a) The quantity of lubricant released from the ERB films at varied mechanical pressures. Digital images of the cotton sheets with the fluorescently labeled lubricant absorbed from the ERB film under different mechanical stimulation are shown. They are illuminated by (b) visible light, and (c) an ultraviolet lamp with a wavelength of 365 nm, respectively. d) The output voltage and (e) short-circuit current measured by friction between PT/SiO_2_ and Cu under different mechanical stimuli (1 N, 3 N, 5 N, 7 N, 10 N, 15 N, 20 N). f) The output voltage and (g) short-circuit current measured by friction between an ERB film and Cu under different mechanical stimuli (1 N, 3 N, 5 N, 7 N, 10 N, 15 N, 20 N).

**
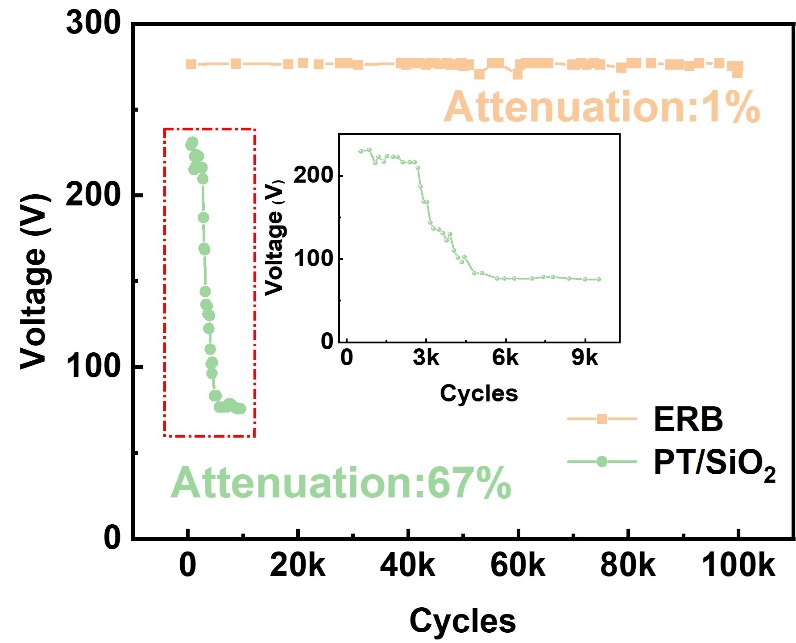
**

**Figure S14.** Comparison of output stability of the PT/SiO_2_ and ERB after continuous sliding 100, 000 cycles.


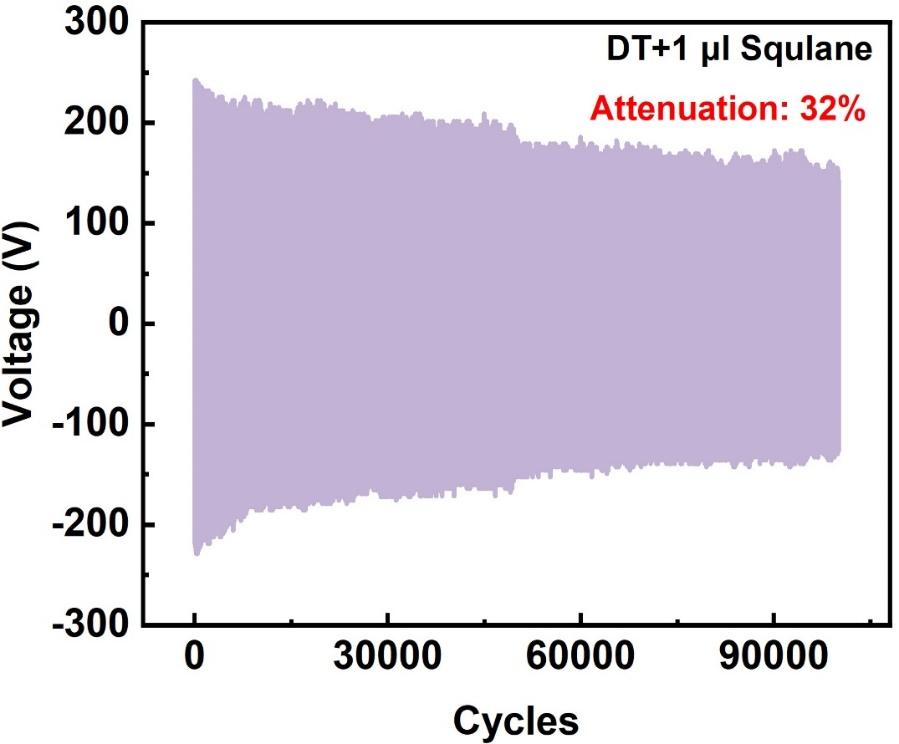


**Figure S15.** Long-term stability of conventional sliding-freestanding TENG based on DT film lubricated by 1 μL squalane after continuous 100,000 cycles.

**
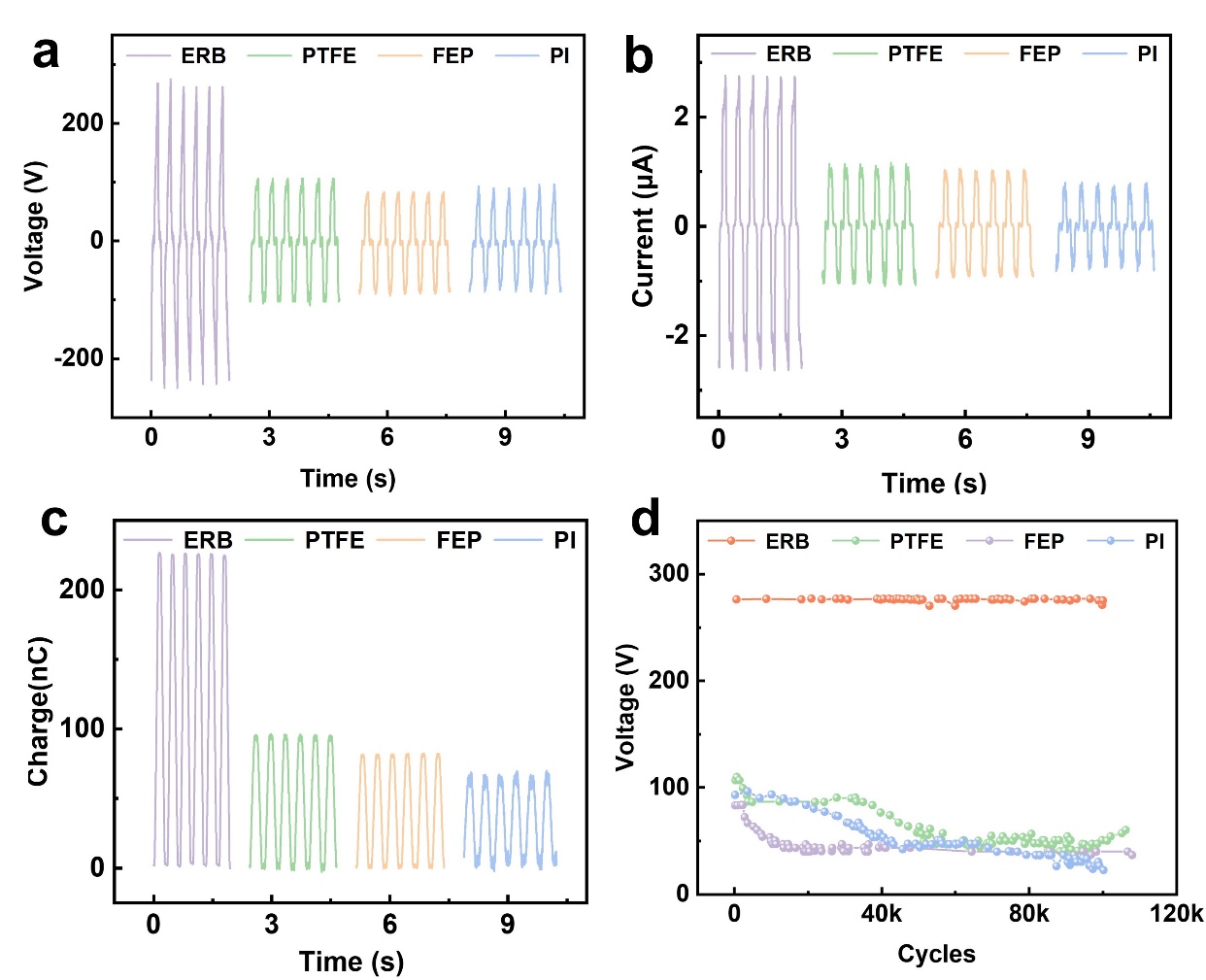
**

**Figure S16.** Comparisons of (a) output voltage, (b) short-circuit current, (c) transferred charge and (d) output stability over 100, 000 continuous sliding friction cycles of ERB, PTFE, FEP and PI.

**
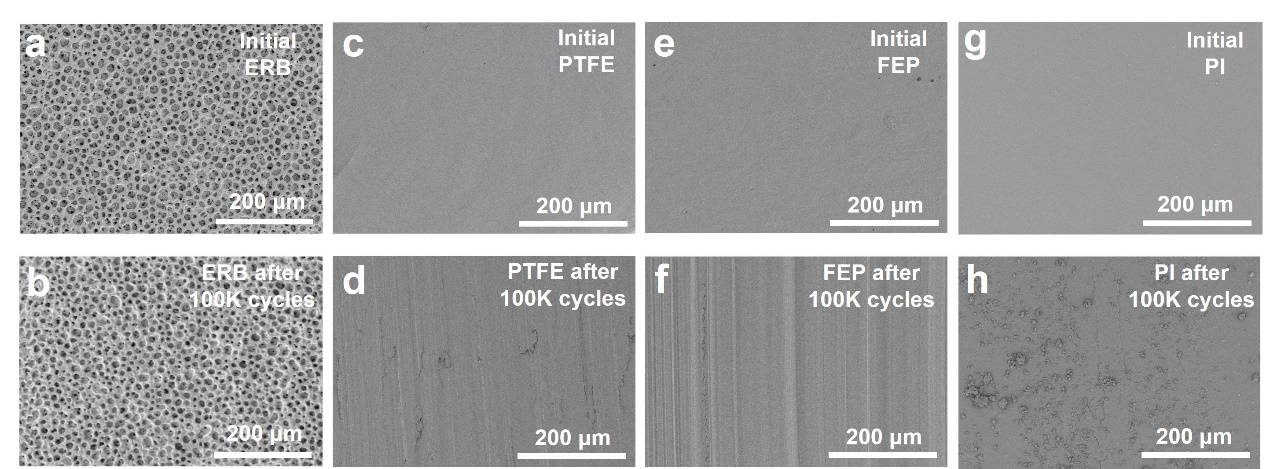
**

**Figure S17.** Surface morphology variation of ERB, PTFE, FEP and PI before and after 100,000 sliding friction cycles.


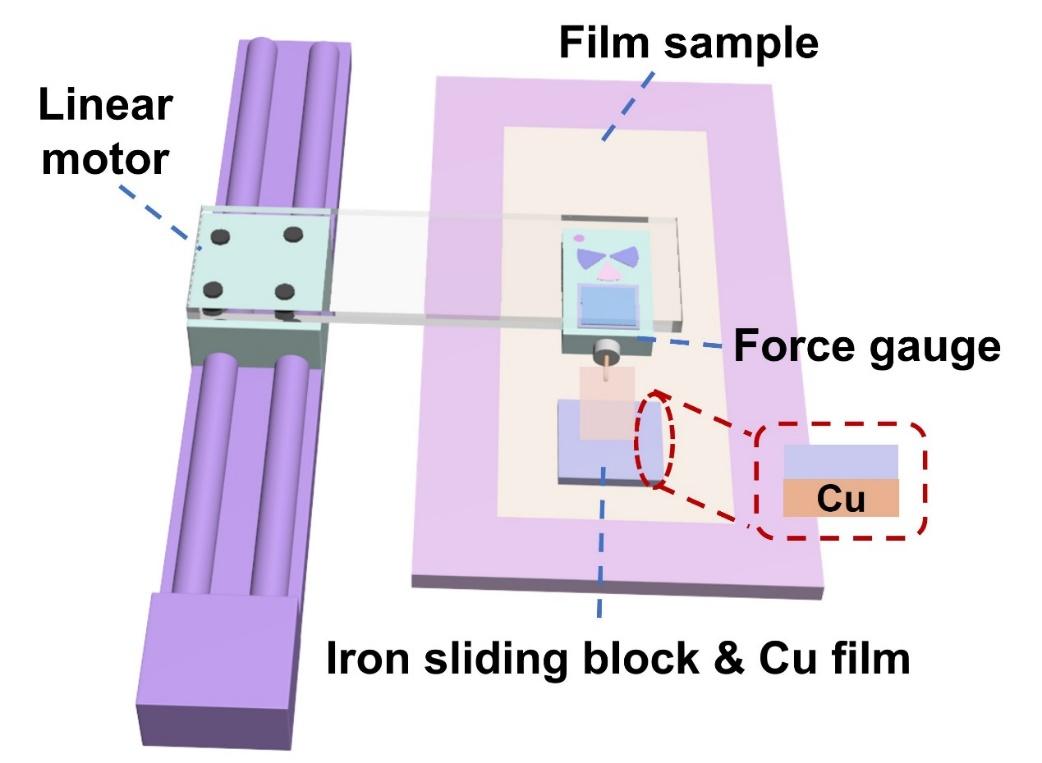


**Figure S18.** Schematic illustration of the dynamic sliding friction test platform.


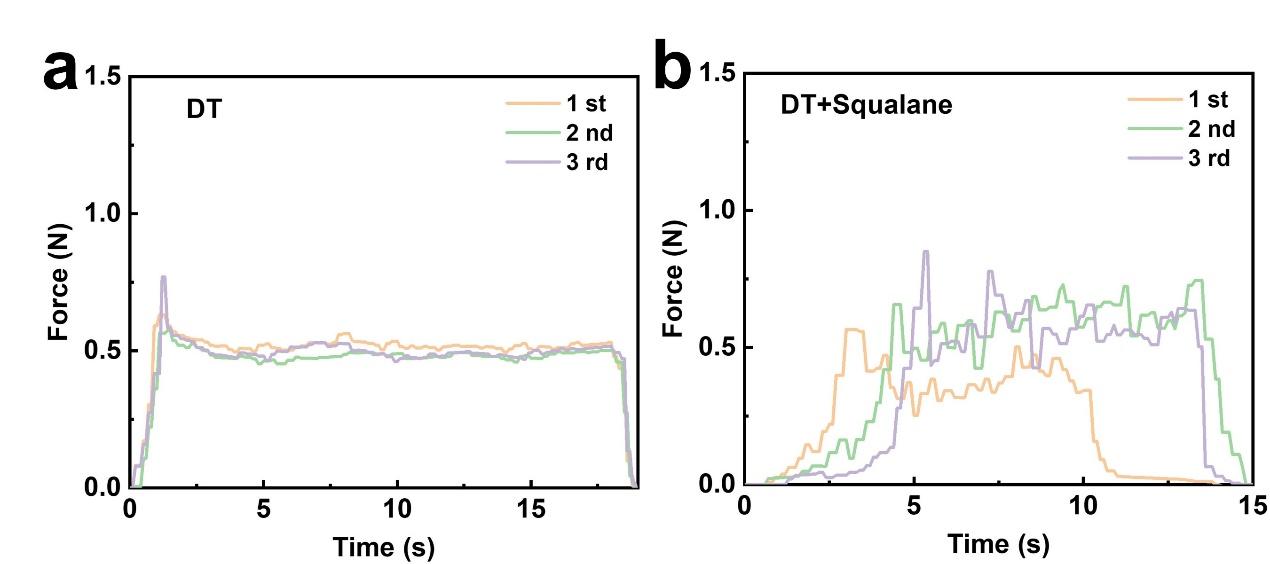


**Figure S19.** The frictional forces were measured under the condition of DT-Cu tribo-pair (a) without lubricant and (b) dripping 200 μL lubricant.

**
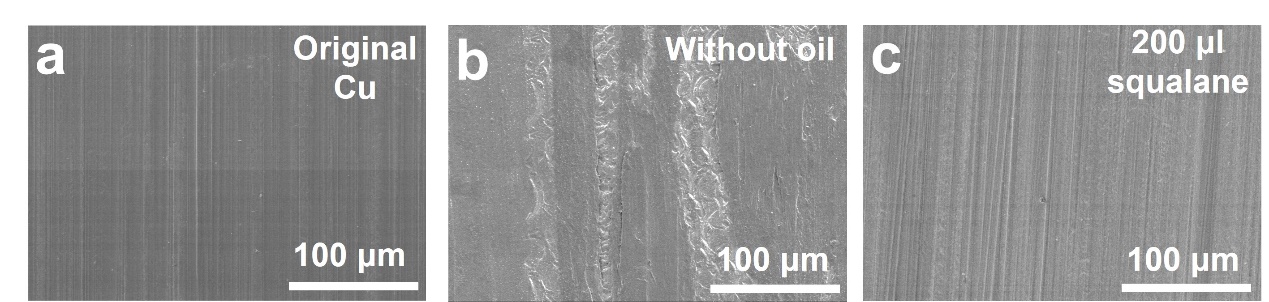
**

**Figure S20.** a) The SEM images of initial Cu foils; b-c) SEM images of Cu foil rubbing against DT (b) without oil and (c) in oil conditions (200 μL squalane) after long-term 100,000 operation cycles, respectively.


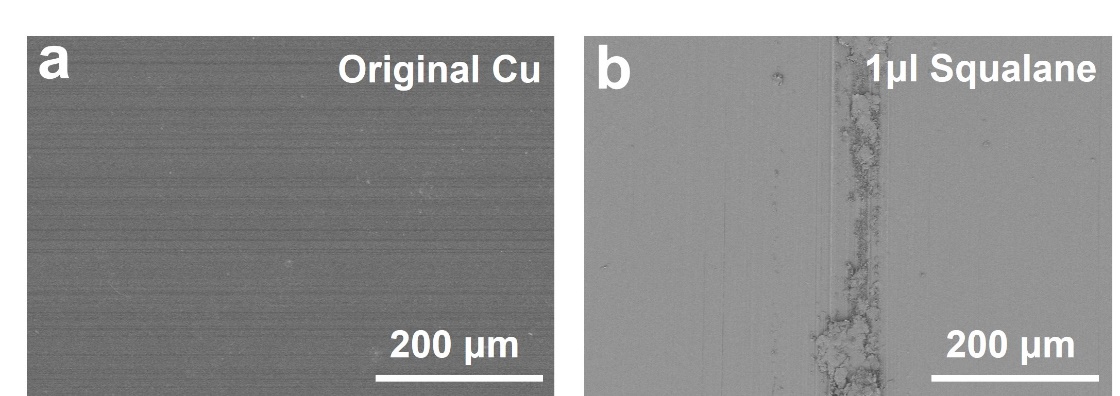


**Figure S21.** SEM images of (a) initial Cu and (b) Cu rubbing against DT lubricated by 1 μL squalane after 100,000 cycles.

**
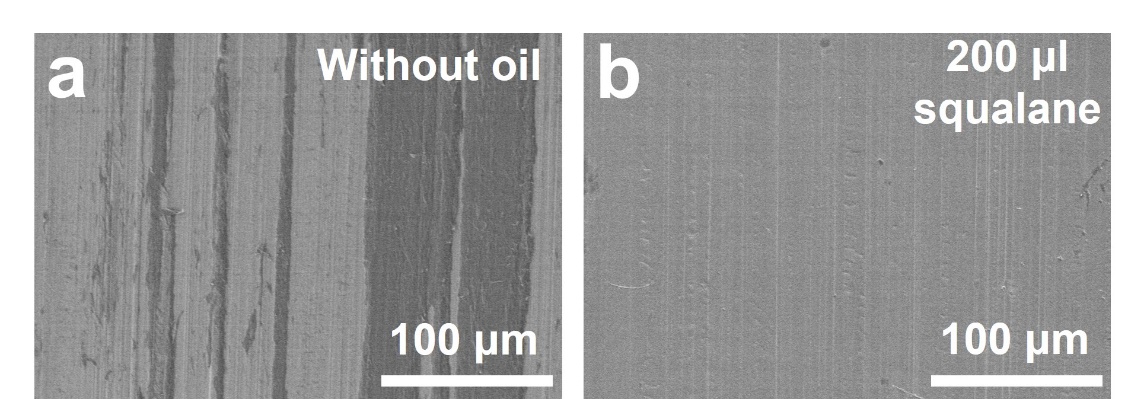
**

**Figure S22.** SEM images of Cu rubbing against PT/SiO_2_ (a) without oil and (b) in oil, respectively, after continuous 100,000 cycles.


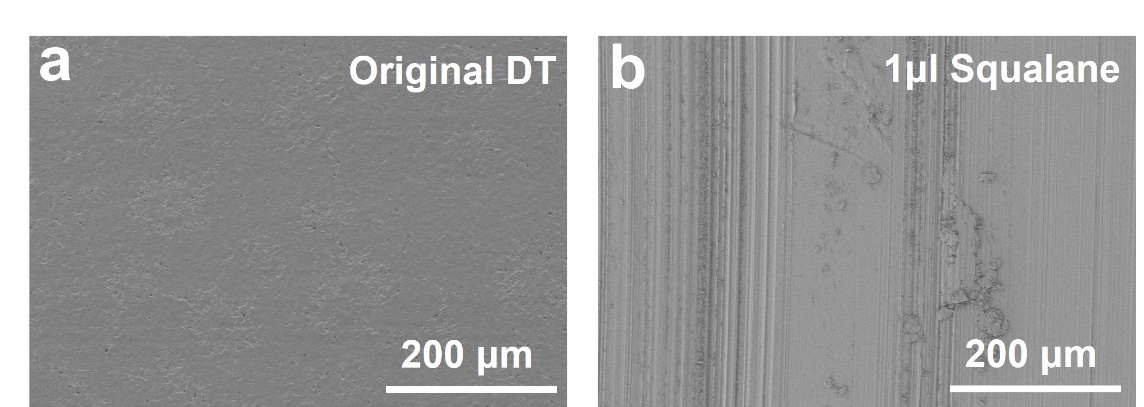


**Figure S23.** SEM images of (a) original DT and (b) DT rubbing with Cu lubricated by 1 μL squalane for 100,000 cycles.


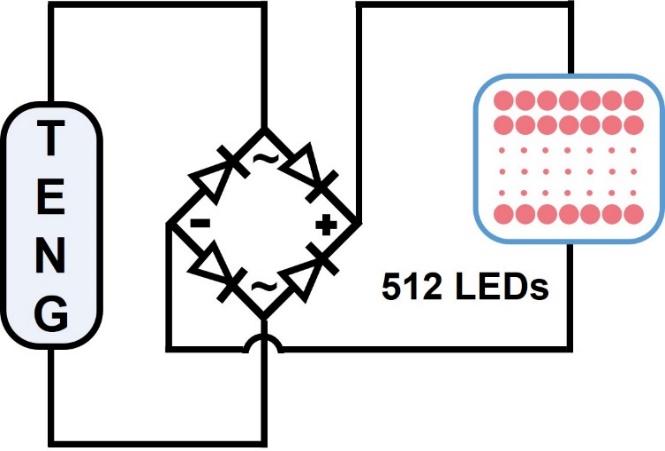


**Figure S24.** Schematic circuit diagram for the simultaneous illumination of the 512 red LEDs connected in sequence.


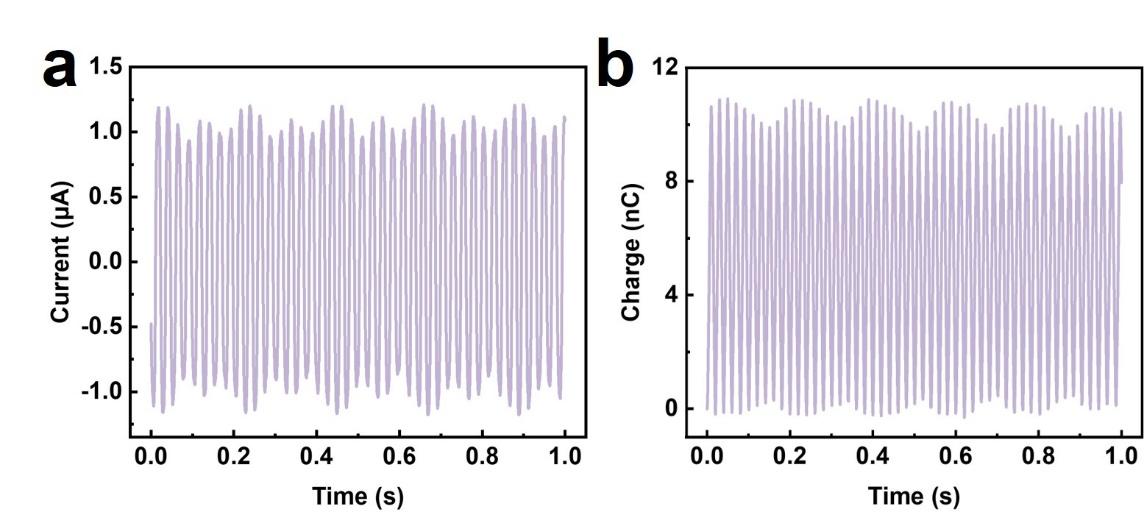


**Figure S25.** The (a) I_sc_ and (b) Q_sc_ of the ER-TENG were obtained at the rotating speed of 270 rpm by harvesting wind energy.

**References**

[1] P. Lu, Y. Xia, *Langmuir* **2013**, *29*, 7070.

[2] B. Zaarour, W. Zhang, L. Zhu, X. Y. Jin, C. Huang, *Text. Res. J.* **2019**, *89*, 2406.

[3] J. Huang, B. Xu, Y. Gao, C. Jiang, X. Guan, Z. Li, J. Han, K. Yan Chung, *Chem. Eng. J.* **2022**, *446*, 137192.

[4] A. Zhang, H. Bai, L. Li, *Chem. Rev.* **2015**, *115*, 9801.

[5] D. Miao, N. Cheng, X. Wang, J. Yu, B. Ding, *Chem. Eng. J.* **2022**, *450*, 138012.
